# Multivariate edaphic gradients are associated with phytochemical quality variation in *Atractylodes chinensis* across northern China

**DOI:** 10.3389/fpls.2026.1962243

**Published:** 2026-10-05

**Authors:** Bo-wen Li, Hai-bo Yin, Dan Wang, Xue-lin Yu, Jing-jie Ma

**Affiliations:** College of Pharmacy, Liaoning University of Traditional Chinese Medicine, Dalian, China

**Keywords:** *Atractylodes chinensis*, false discovery rate, HPLC, medicinal quality, principal component analysis, secondary metabolites, soil nutrients

## Abstract

Environmental heterogeneity is widely invoked to explain geographic variation in medicinal plant quality. However, inference based on numerous intercorrelated soil variables can be unstable when individual correlations are interpreted separately. In this study, 42 rhizome–soil paired samples of *Atractylodes chinensis* were purposively selected across northern China to capture phytochemical variation and associated environmental data. The dataset included 32 cultivated samples and 10 wild reference samples; within Liaoning Province, the sampling set contained 25 cultivated samples and seven wild reference samples from corresponding production and distribution areas. Seven targeted phytochemical markers were quantified by HPLC, and 13 soil physicochemical variables were measured. Principal component analysis (PCA) was conducted separately on phytochemical and soil datasets. Using the Kaiser criterion (eigenvalue >1), six soil principal components (PCs) and two chemical PCs were retained, and all 12 cross-space PC combinations were tested with BH-FDR correction. Chemical PC1 and PC2 explained 37.68% and 23.22% of the total variance in phytochemical profiles, respectively. Soil PC1 and PC2 were not significantly associated with chemical PC1 (*r*=0.019, *P* = 0.906 and *r*=0.249, *P* = 0.112, respectively), whereas soil PC3 was significantly associated with chemical PC1 (Pearson *r*=0.465, *P* = 0.0019, q=0.0229; Spearman *r*=0.459, *P* = 0.0022; *R*^2^=0.216; non-parametric bootstrap 95% CI: 0.24–0.66). This association remained significant in cultivated samples (*n*=32, *r*=0.538, *P* = 0.0015), Liaoning samples (*n*=32, *r*=0.570, *P* = 0.00066), and the 25 Liaoning cultivated samples (*r*=0.630, *P* = 0.00074), while no significant association was observed in the small wild subset (*n*=10, *r*=0.196, *P* = 0.587). A sensitivity analysis controlling for four retained climate PCs plus altitude yielded a similar partial association across the full dataset (partial *r*=0.484, *P* = 0.0024). This study identifies a reproducible multivariate association between soil properties and phytochemical profiles in cultivated *A. chinensis*. Nevertheless, purposive sampling, unknown genotype information, and the observational design preclude causal attribution to any individual soil factor.

## 1 Introduction

Atractylodis Rhizoma is the dried rhizome of *Atractylodes lancea* (Thunb.) DC. or *Atractylodes chinensis* (DC.) Koidz. and is an important traditional medicinal material in East Asia (Chinese Pharmacopoeia Commission, 2020; Zhang WJ. et al., 2021; Sun et al., 2026). Its chemical composition is complex and includes sesquiterpenes, polyacetylenes, lactones and other classes of specialized metabolites (Zhang WJ. et al., 2021; Xing et al., 2025; Zhang et al., 2025; Sun et al., 2026). For *A. chinensis*, atractylodin, atractylon, β-eudesmol and hinesol are among the characteristic volatile or semi-volatile constituents used in chemical evaluation, while atractylenolides and other components contribute additional dimensions of phytochemical quality (Zhang WJ. et al., 2021; Zhao et al., 2023; Xing et al., 2025; Zhang et al., 2025; Sun et al., 2026). Because medicinal materials are harvested as biologically heterogeneous plant organs rather than purified chemicals, quality is more appropriately viewed as a multivariate phenotype than as the concentration of a single marker (Sun et al., 2021b; Zhao et al., 2023; Ji et al., 2024).

Geographic origin is a major source of variation in Atractylodes chemistry. In *A. lancea*, common-garden experiments showed substantial genetic contributions to variation in β-eudesmol, hinesol, atractylon and atractylodin, while environmental differences also altered chemical profiles (Sun et al., 2021a; Tsusaka et al., 2019). Metabolite-marker approaches have further demonstrated strong chemotypic differentiation among geographic origins (Zhang et al., 2023). Recent studies of *A. chinensis* likewise emphasize provenance-dependent variation in atractylodin and other active ingredients (Bai et al., 2025; Lu et al., 2026; Wang et al., 2026; You et al., 2026). These observations are consistent with the broader concept that medicinal-plant quality emerges from interactions among genotype, development, edaphic conditions, climate and management rather than from one environmental variable acting in isolation (Ramakrishna and Ravishankar, 2011; Isah, 2019; Applequist et al., 2020; Pant et al., 2021; Zhang S. et al., 2021; Jangpangi et al., 2025; You et al., 2026).

Soil is especially relevant because it integrates nutrient supply, pH, organic matter, water relations and rhizosphere biology. Nitrogen, phosphorus and potassium support primary metabolism and biomass production, whereas micronutrients and soil organic matter can alter enzyme activity, redox balance, carbon allocation and nutrient availability. At the same time, soil properties shape rhizosphere microbial communities that participate in nutrient cycling and plant stress responses (van der Heijden et al., 2008; Bulgarelli et al., 2013; Mendes et al., 2013; Fierer, 2017). In Atractylodes, continuous cropping and rhizosphere changes have been associated with altered soil chemistry, autotoxicity and microbial composition (Tang et al., 2023; Guo et al., 2005; Wang et al., 2023). Geographic comparisons of *A. lancea* and *A. chinensis* have also linked active-ingredient variation with soil nutrients and microbiome structure (Tang et al., 2023; Bai et al., 2025; Lu et al., 2026). These studies make soil a biologically plausible source of quality variation, but they do not imply that each correlated soil element independently causes a change in a particular metabolite.

Environmental regulation of secondary metabolism is inherently multivariate. Water deficit can reprogram photosynthesis, antioxidant capacity and specialized metabolism in Atractylodes (Zhang A. et al., 2021; Ma et al., 2024; Wu et al., 2026), and recent irrigation experiments in *A. chinensis* have shown that water supply simultaneously influences plant structure, yield and quality (Lv et al., 2026). Light quality, light intensity, temperature and other abiotic stresses can also modify secondary-metabolite biosynthesis in medicinal plants (Ramakrishna and Ravishankar, 2011; Isah, 2019; Applequist et al., 2020; Pant et al., 2021; Zhang S. et al., 2021; Jangpangi et al., 2025). Importantly, the direction and magnitude of responses differ among compounds and developmental contexts. Therefore, an ecological-quality study that tests dozens of soil-metabolite correlations without controlling the multiplicity of tests risks identifying unstable associations that are difficult to reproduce.

The original dataset underlying the present study comprised paired rhizome and soil samples collected across northern China and contained 42 samples with seven targeted compounds and 13 measured soil variables. Earlier analyses emphasized individual correlations and path coefficients. We revisited the complete sample-level soil and phytochemical matrices to develop a more conservative and reproducible statistical framework. Our objectives were to (i) characterize multi-marker phytochemical variation across the 42 samples; (ii) quantify edaphic heterogeneity and reduce correlated soil variables to interpretable multivariate axes; (iii) test all soil-marker associations with explicit false-discovery-rate control; and (iv) determine whether any cross-space association was robust to the dominant geographic sampling region and to cultivated-versus-wild production mode. We hypothesized that coordinated phytochemical variation would be more strongly associated with a composite soil nutrient gradient than with any single edaphic variable. This formulation treats the field data as observational evidence of covariation and generates hypotheses for subsequent controlled cultivation experiments rather than claiming direct causal effects.

## 2 Materials and methods

### 2.1 Study design, sample provenance and field context

The field program was conducted during 2022–2023 in major *A. chinensis* production and distribution areas of northern China. The 42 analytical samples were not a random or systematic subset of the broader 69-site resource survey. They were purposively selected from archived materials for which both rhizome and paired soil measurements were available. Within Liaoning, 25 cultivated samples were selected to span the observed low-to-high phytochemical profiles across major cultivation localities, and seven wild samples from corresponding cities or nearby natural distribution areas were included as reference materials. Additional samples were included from Hebei and Inner Mongolia because both regions contain natural populations and are major medicinal-material production areas, whereas the Jilin and Heilongjiang sampling frame contained cultivated bases only. The final analytical dataset therefore comprised 32 cultivated and 10 wild reference samples from Liaoning (n=32), Hebei (n=4), Jilin (n=3), Heilongjiang (n=1), and Inner Mongolia (n=2). Inclusion required species authentication, sufficient rhizome material for HPLC analysis, and a complete paired soil sample; samples lacking paired analytical material were excluded. Exact latitude, longitude, and altitude for all 42 analytical samples were recovered from the field records and are provided in **Supplementary Table S12**. Plant material was authenticated as *A. chinensis* by Professor Haibo Yin. Representative cultivated and wild morphologies are provided in **Supplementary Figure S2** as supporting documentation of species-level authentication; these photographs are not intended to resolve within-species genetic variation. The broader 69-site survey is reported separately in **Supplementary Table S14** and should be regarded as geographic context rather than a one-to-one list of the 42 analytical vouchers.

### 2.2 Rhizome preparation and HPLC determination

Rhizomes were washed, dried and powdered through an 80-mesh sieve. Powder (0.5 g) was transferred to a stoppered conical flask, mixed with 20 mL methanol, protected from light and sonicated for 30 min at 25 ± 1 °C (240 W, 40 kHz). After cooling, methanol was added to compensate for weight loss, and the extract was mixed, filtered and passed through a 0.45 μm membrane. Seven compounds were quantified with an Agilent 1260 HPLC system equipped with a C18 column (250 mm × 4.6 mm, 5 μm; Dikma Diamonsil RP18E). The mobile phase was acetonitrile (A) and water (B): 0–10 min, 10-15% A; 10–15 min, 15-55% A; 15–45 min, 55-90% A; 45–50 min, 90-100% A; and 50–55 min, 100-10% A. Column temperature was 30 °C, flow rate 0.8 mL min−1, injection volume 10 μL and detection wavelength 203 nm. The markers were atractylon, atractylodin, β-eudesmol, hinesol, atractylenolide I, atractylenolide III and 5-HMF. The independent ecological sample size was n=42.

### 2.3 HPLC method validation

Calibration was evaluated by injecting a mixed reference solution at 1, 2, 4, 6, 8, 10 and 20 μL. Precision was assessed by six consecutive injections of one sample solution; stability at 0, 2, 4, 8, 12 and 24 h; repeatability using six independently prepared aliquots; and recovery using six spiked samples. Calibration equations, linear ranges and validation statistics are provided in **Supplementary Table S2**, and archived chromatograms for linearity, precision, repeatability, stability, spike recovery and representative sample analysis are shown in **Supplementary Figure S1**. The archived files did not retain low-level replicate measurements, baseline noise values, or signal-to-noise data sufficient for defensible retrospective estimation of LOD and LOQ. We therefore describe the method as validated for linearity, precision, stability, repeatability and recovery, rather than as a fully validated analytical method with established detection and quantification limits.

### 2.4 Soil physicochemical measurements

Air-dried soil was ground and passed through an 80-mesh sieve. Thirteen variables were measured: pH, organic matter, alkali-hydrolyzed nitrogen, available phosphorus, available potassium, available boron, calcium, magnesium, iron, manganese, zinc, copper and molybdenum. Soil pH was determined potentiometrically at a water:soil ratio of 2.5:1; organic matter by potassium dichromate volumetry with external heating; alkali-hydrolyzed nitrogen by alkaline diffusion; available phosphorus by bicarbonate extraction followed by molybdenum-antimony colorimetry; available boron by hot-water extraction and curcumin colorimetry; available potassium by ammonium acetate extraction and atomic absorption; and Ca, Mg, Fe, Mn, Zn, Cu and Mo by alkaline fusion followed by inductively coupled plasma optical emission spectrometry. The source statement of 14 soil factors was a counting error; the analytical matrix contains 13 variables.

### 2.5 Statistical analysis

All reanalyses used the recovered sample-level 42 × 7 chemical matrix and 42 × 13 soil matrix. Chemical variables were log1p transformed because most raw concentrations were right-skewed; the median absolute skewness across the seven markers decreased from 1.44 before transformation to 0.70 after transformation. The transformation was used to reduce leverage of extreme concentrations rather than to force exact normality. All variables were then standardized to zero mean and unit variance before PCA (Jolliffe and Cadima, 2016). Scree plots were inspected and the Kaiser criterion (eigenvalue >1) was used as the explicit component-retention rule. This retained chemical PC1-PC2 and soil PC1-PC6. All 12 retained soil-PC × chemical-PC combinations were tested rather than selecting soil PC3 *a priori*, and BH-FDR correction was applied across the 12 tests. Pairwise relationships between the 13 original soil variables and seven chemical markers were evaluated with Spearman correlation (91 tests) because several original variables were skewed and contained influential values, followed by BH-FDR correction. For PC-score associations, Pearson correlations were reported because PCA defines linear score axes; however, Shapiro-Wilk testing indicated approximate normality for chemical PC1 (P = 0.123) but not for soil PC3 (P = 0.007), so Spearman coefficients were additionally reported as a robustness check for every retained PC pair. The main association was accepted only when Pearson and Spearman analyses agreed in direction and inference. Non-parametric case-resampling bootstrap confidence intervals used 10,000 resamples and percentile 95% limits (Efron and Tibshirani, 1993). Sensitivity analyses were performed for cultivated samples, all Liaoning samples, the 25 Liaoning cultivated samples, and the small wild reference subset. For interpretive context, the full sample size of n=42 provides approximately 80% power to detect |r|≈0.41 and approximately 90% power for the observed r≈0.47 at two-sided α=0.05; smaller effects and the n=10 wild subset remain underpowered. Analyses were implemented in Python 3.13.5 using NumPy 2.3.5, SciPy 1.17.0, scikit-learn 1.8.0, statsmodels 0.14.6 and Matplotlib 3.10.8.

### 2.6 Archived geography and climate information

A complete sample-level environmental workbook was recovered during revision. It contains exact coordinates, altitude, 19 standard BIOCLIM variables, monthly precipitation and temperature summaries, and additional site descriptors for the 42 analytical samples. To avoid overfitting a highly collinear climate matrix, the 19 BIOCLIM variables were standardized and summarized by PCA; the first four climate PCs had eigenvalues >1 and together explained 98.17% of climatic variance. A prespecified sensitivity analysis estimated the partial correlation between soil PC3 and chemical PC1 after residualizing both axes against climate PC1-PC4 and standardized altitude. Direct measurements of soil moisture/hydrological flux, rhizosphere microbiome composition, and molecular genotype were not available and are treated as unresolved confounders rather than modeled covariates.

## 3 Results

### 3.1 Sampling structure and analytical performance

The analytical dataset contained 42 paired rhizome-soil samples from five provincial-level regions (**Figures 1A, B**). The sampling design was purposive rather than regionally balanced (**Figure 1C**): Liaoning contributed 25 cultivated samples selected to span phytochemical variation and seven wild reference samples from corresponding distribution areas; the remaining ten samples comprised seven cultivated and three wild references from Hebei, Jilin, Heilongjiang and Inner Mongolia. Thus, the final analytical set contained 32 cultivated and 10 wild reference samples. The exact GPS coordinates of the analytical vouchers are now shown in **Figure 1A** and **Supplementary Table S12**. Because the principal objective was to evaluate environmental covariation in cultivated materials rather than estimate province-level means, province was retained only as a descriptive label. Representative cultivated and wild morphologies are shown in **Supplementary Figure S2**.

**Figure 1:**
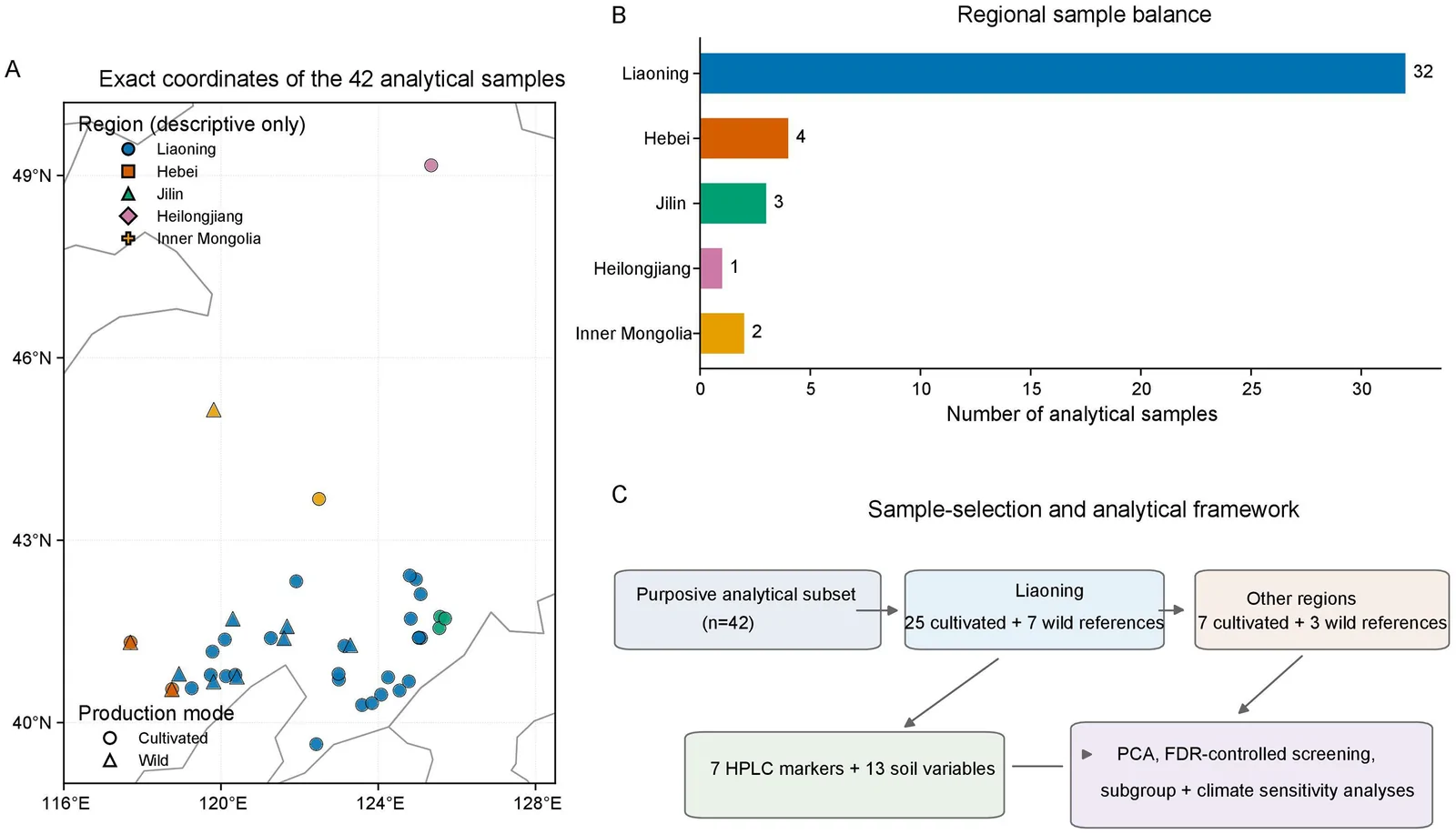
Sampling framework and analytical design. **(A)** Exact coordinates of the 42 analytical samples. All points are plotted at fixed size; region is encoded with a colorblind-accessible color/shape scheme and production mode is indicated by point shape. Regional coding is descriptive only. **(B)** Regional sample counts. **(C)** Purposive sample-selection framework: 25 cultivated and seven wild reference samples from Liaoning plus ten samples from other regions, followed by HPLC, soil, PCA, FDR-controlled screening and climate sensitivity analyses.

The HPLC validation results supported quantitative comparison among samples. Calibration coefficients ranged from R²=0.9991 to 0.9996. Precision RSDs were 1.315-2.946%, 24-h stability RSDs were 1.207-2.924%, and repeatability RSDs were 0.944-2.828%. Mean spike recoveries ranged from 95.512% to 103.053%, with recovery RSDs of 1.388-3.857% (**Supplementary Table S2**). The archived chromatograms consistently contained the seven annotated marker peaks across the linearity, precision, repeatability, stability, recovery and sample runs (**Supplementary Figure S1**).

The reported linear ranges were 1.20-11.9 μg for atractylon, 1.05-10.5 μg for atractylodin, 0.94-9.4 μg for β-eudesmol, 1.26-12.6 μg for hinesol, 0.93-9.3 μg for atractylenolide I, 0.58-5.8 μg for atractylenolide III and 0.40-4.0 μg for 5-HMF. Across the validation experiments, no analyte showed an RSD above 3% for precision, stability or repeatability. The spike-recovery means were distributed around 100%, with the lowest mean for hinesol (95.512%) and the highest for atractylenolide III (103.053%).

### 3.2 Phytochemical composition showed marked multi-marker heterogeneity

The seven targeted compounds varied substantially across the 42 samples (**Figure 2A**; **Supplementary Tables S3, S8**). Atractylon ranged from 0.500 to 23.876 mg g−1 (CV 87.5%), β-eudesmol showed a CV of 91.9%, and hinesol a CV of 69.1%. Atractylenolide I showed the greatest relative dispersion among the seven markers (CV 112.2%), whereas atractylodin was comparatively less variable (CV 36.6%). The standardized heat map was redrawn with non-overlapping voucher labels and showed that high or low concentrations commonly occurred across several compounds within the same sample rather than being restricted to one analyte.

**Figure 2:**
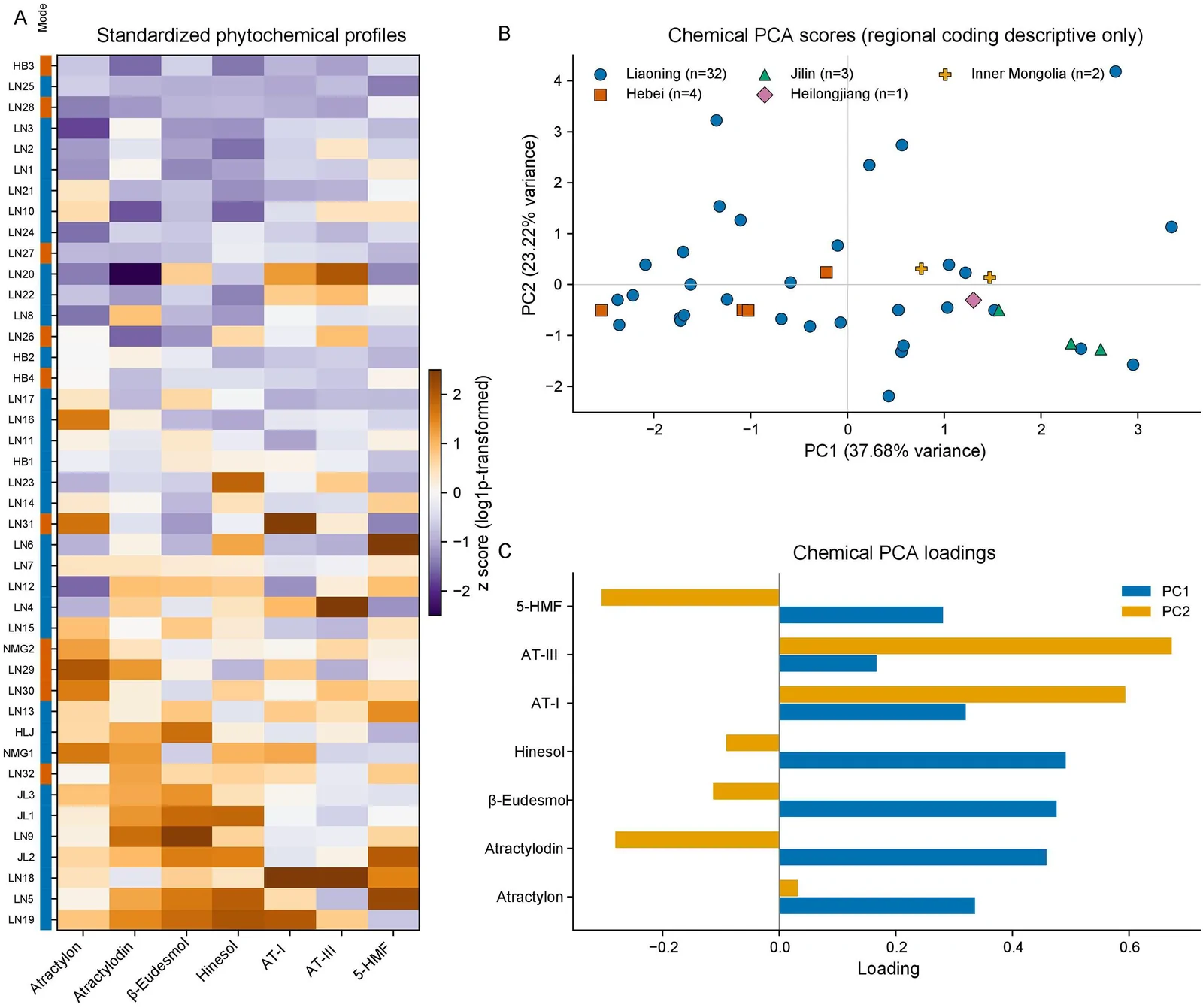
Phytochemical heterogeneity across 42 *A. chinensis* samples. **(A)** Heat map of log1p-transformed and standardized concentrations ordered by chemical PC1; voucher labels were reformatted to avoid overlap and the side strip denotes production mode. **(B)** Chemical PCA scores with colorblind-accessible regional coding; region is shown descriptively only because sampling was unbalanced. **(C)** Loadings of the seven markers on PC1 and PC2.

Chemical PCA captured this coordinated variation. PC1 explained 37.68% and PC2 explained 23.22% of total chemical variance, for a cumulative 60.90% (**Figure 2B**). These were the only chemical PCs with eigenvalues >1 (2.70 and 1.66, respectively) and were therefore retained under the prespecified Kaiser criterion. All seven compounds loaded positively on chemical PC1, with the largest loadings from hinesol (0.491), β-eudesmol (0.476) and atractylodin (0.458); chemical PC1 was therefore interpreted as a coordinated multi-marker abundance axis (**Figure 2C**). Samples from different regions overlapped broadly in the PC1-PC2 score space. Regional colors and symbols in **Figure 2B** are descriptive only and are not intended to support formal regional differentiation.

The largest chemical PC1 scores occurred in LN19, LN5, LN18, JL2 and LN9, whereas HB3, LN25, LN28, LN3 and LN2 occupied the lower end of the axis. This ordering was not restricted to a single province or production mode. The raw concentration distributions were also compound specific: atractylon reached 23.876 mg g−1, atractylenolide I reached 11.055 mg g−1, and 5-HMF remained quantitatively lower, ranging from 0.022 to 0.788 mg g−1. These differences in scale were removed by transformation and standardization before PCA.

### 3.3 Paired soils encompassed broad and multidimensional edaphic variation

The 13 measured soil properties also varied widely among the paired samples (**Figure 3A**; **Supplementary Table S4**). Soil pH ranged from 4.62 to 8.44 and showed the lowest coefficient of variation (14.8%). In contrast, manganese (CV 193.0%), available phosphorus (CV 130.8%) and calcium (CV 122.0%) were highly heterogeneous. Organic matter and alkali-hydrolyzed nitrogen also varied substantially, with CVs of 54.6% and 51.3%, respectively.

**Figure 3:**
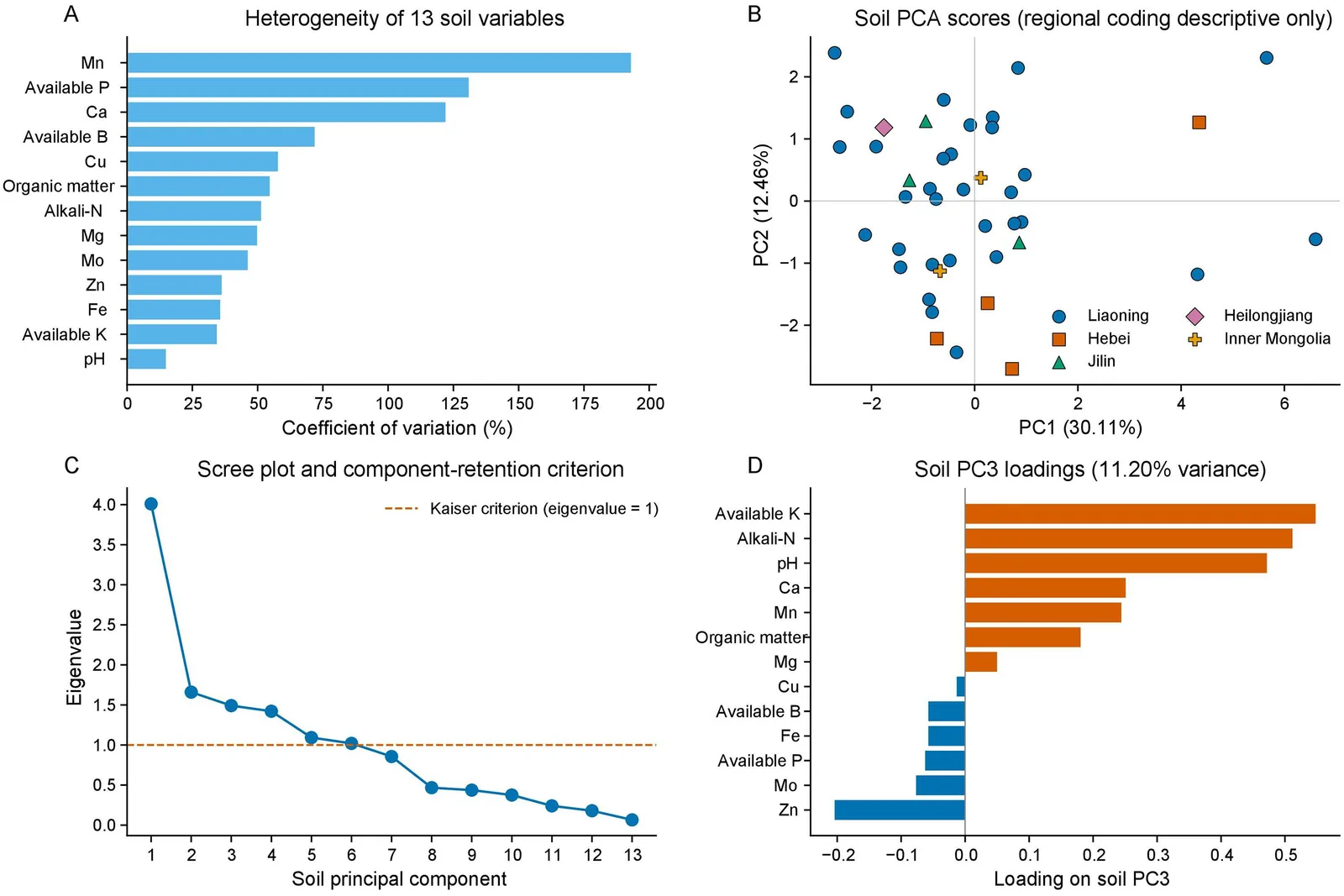
Edaphic heterogeneity and PCA retention. **(A)** Coefficients of variation for 13 soil variables. **(B)** Soil PC1-PC2 scores with region shown descriptively only. **(C)** Scree plot with the Kaiser eigenvalue=1 retention threshold; soil PC1-PC6 were retained. **(D)** Loadings on soil PC3.

The first six standardized soil PCs all had eigenvalues >1 and together accounted for 80.31% of total edaphic variance (**Figures 3B, C**; **Supplementary Table S10**). Specifically, soil PC1-PC6 explained 30.11%, 12.46%, 11.20%, 10.67%, 8.20% and 7.66%, respectively. The scree plot and the Kaiser criterion were used for retention; soil PC3 was therefore one of six retained axes rather than being selected because of its subsequent association result. Soil PC3 was defined most strongly by positive loadings for available K (0.548), alkali-hydrolyzed N (0.512) and pH (0.472), with additional positive contributions from Ca and Mn (**Figure 3D**). The complete loadings for all soil PCs are provided in **Supplementary Table S7**.

Soil PC3 scores extended across a broad continuous range, and no province formed a discrete cluster on the retained axes. The underlying soil variables likewise covered large absolute ranges, including available phosphorus from 2.70 to 951.20 mg kg−1, calcium from 0.49 to 80.74 g kg−1 and manganese from 280 to 10,087 mg kg−1. The PCA therefore summarized broad edaphic heterogeneity, but the similar eigenvalues of soil PC2-PC4 also reinforce the need to report the complete retained-PC screening rather than emphasizing one component without multiplicity control.

### 3.4 Individual soil-marker correlations did not remain significant after FDR correction

All 13 × 7 soil-marker combinations were tested, giving 91 pairwise Spearman correlations (**Figure 4A**; **Supplementary Table S15**). Seven associations reached nominal P<0.05, but none remained significant after Benjamini-Hochberg FDR correction (**Figure 4B**). The smallest unadjusted P value occurred for organic matter versus atractylon (ρ=0.436, P = 0.0039), followed by alkali-hydrolyzed N versus hinesol (ρ=0.432, P = 0.0043); both adjusted q values were 0.194. The nominal signals were distributed across several soil variables and markers rather than converging on one repeated soil-chemical relationship.

**Figure 4:**
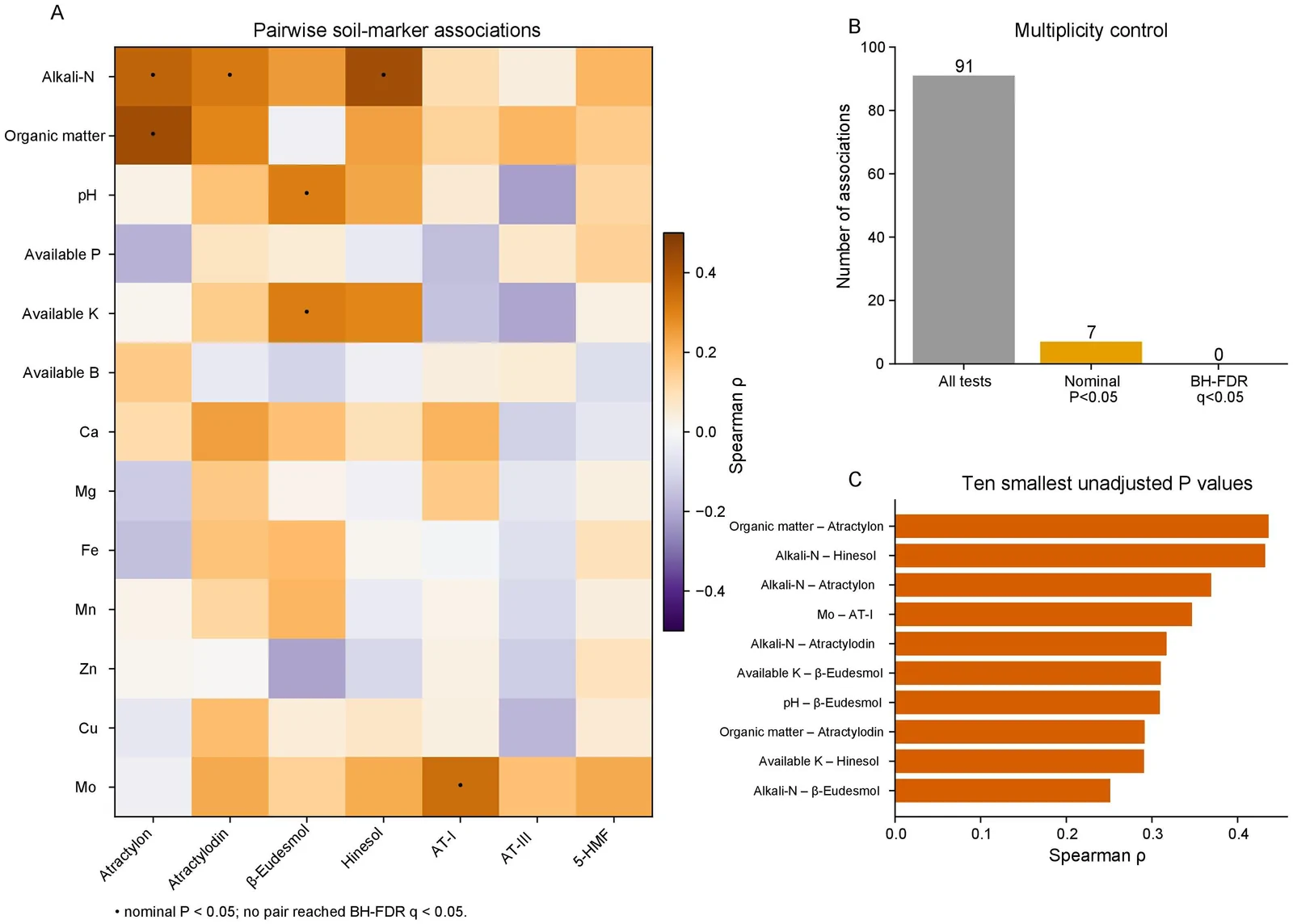
Pairwise soil-marker association analysis. **(A)** Colorblind-accessible Spearman correlation heat map for 13 soil variables and seven phytochemical markers; bullets denote nominal P<0.05. **(B)** Number of associations before and after multiplicity control. **(C)** Ten associations with the smallest unadjusted P values. Complete ρ, P and BH-FDR q values are provided in **Supplementary Table S15**; no individual pair reached q<0.05.

Thus, the current dataset provides no FDR-robust evidence that any individual measured soil factor independently predicts a specific targeted compound. The unadjusted associations are retained as descriptive candidates in the supporting data, whereas subsequent inference focuses on multivariate soil and chemical structure.

The seven nominal associations were organic matter-atractylon (ρ=0.436, P = 0.0039), alkali-hydrolyzed N-hinesol (ρ=0.432, P = 0.0043), alkali-hydrolyzed N-atractylon (ρ=0.369, P = 0.0162), Mo-atractylenolide I (ρ=0.347, P = 0.0245), alkali-hydrolyzed N-atractylodin (ρ=0.317, P = 0.0408), available K-β-eudesmol (ρ=0.310, P = 0.0456) and pH-β-eudesmol (ρ=0.309, P = 0.0463). Their adjusted q values ranged from 0.194 to 0.602, so none was retained as an individually significant association (**Figure 4C**).

### 3.5 Soil PC3 was positively associated with the multi-marker chemical PC1 axis

To address component-selection bias explicitly, all 12 combinations formed by the six retained soil PCs and two retained chemical PCs were tested and corrected together (**Figure 5A**; **Supplementary Table S11**). Soil PC1 was not associated with chemical PC1 (r=0.019, P = 0.906, q=0.906), and soil PC2 showed only a weak non-significant association with chemical PC1 (r=0.249, P = 0.112, q=0.671). Soil PC3-chemical PC1 was the only retained-PC pair that remained significant after BH-FDR correction (Pearson r=0.465, P = 0.0019, q=0.0229; **Figure 5B**). Because soil PC3 was not normally distributed by Shapiro-Wilk testing, we also evaluated rank correlation; the result was concordant (Spearman ρ=0.459, P = 0.0022, q=0.0268). Non-parametric bootstrap resampling (10,000 case resamples) gave a percentile 95% CI of 0.24-0.66. The raw association explained 21.6% of the variance in chemical PC1 (R²=0.216), indicating a moderate rather than deterministic effect.

**Figure 5:**
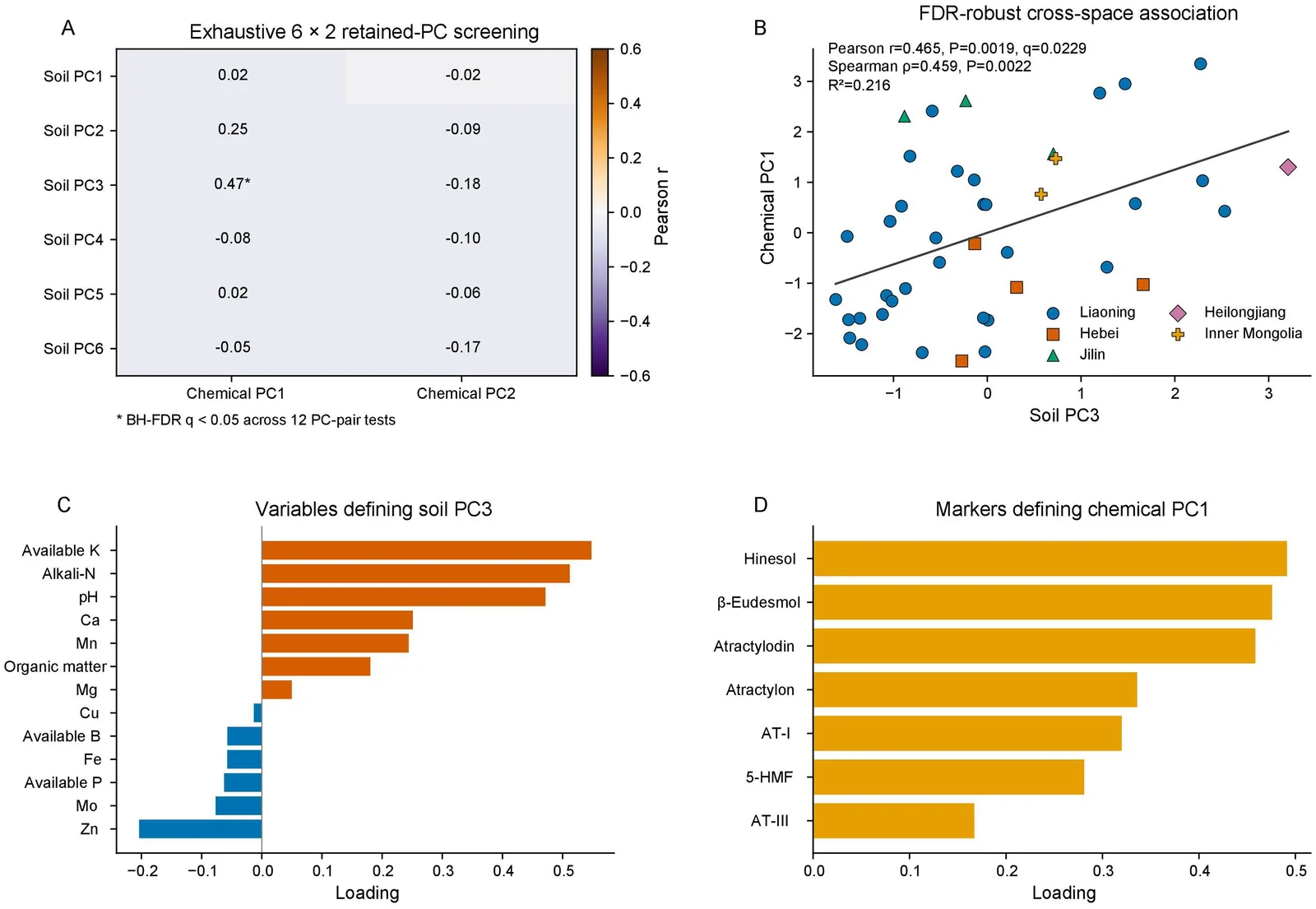
Exhaustive retained-PC screening and the principal cross-space association. **(A)** Pearson correlations for all six retained soil PCs × two retained chemical PCs; * indicates BH-FDR q<0.05 across 12 tests. **(B)** Soil PC3-chemical PC1 association with Pearson and Spearman statistics and R². Regional coding is descriptive only. **(C)** Soil PC3 loadings. **(D)** Chemical PC1 loadings.

The loadings provide a direct description of the associated axes. Soil PC3 was dominated by available K, alkali-hydrolyzed N and pH, with smaller positive contributions from Ca, Mn and organic matter (**Figure 5C**), whereas chemical PC1 had positive contributions from all seven compounds, particularly hinesol, β-eudesmol and atractylodin (**Figure 5D**). Thus, the reproducible signal is a multivariate edaphic axis associated with coordinated phytochemical abundance; the analysis does not identify any single soil factor as a causal driver.

The exhaustive retained-PC matrix also showed why selective reporting was inappropriate: none of the remaining 11 combinations reached FDR significance, and their Pearson coefficients ranged from -0.180 to 0.249. Regional symbols remained intermingled around the fitted soil PC3-chemical PC1 relationship, consistent with a continuous cross-sample association rather than a categorical province effect.

### 3.6 The principal association was reproducible in cultivated and Liaoning subsets and remained after climate adjustment

Production mode did not generate significant shifts in the two axes defining the main association. Chemical PC1 did not differ significantly between cultivated and wild samples (Mann-Whitney P = 0.506), and soil PC3 likewise showed no significant production-mode difference (P = 0.894) (**Figures 6A, B**). When analyzed separately, cultivated samples retained a positive soil PC3-chemical PC1 relationship (n=32, r=0.538, P = 0.0015). The small wild reference subset showed only a weak, non-significant relationship (n=10, r=0.196, P = 0.587) and is therefore treated as descriptive rather than as independent validation (**Figure 6C**).

**Figure 6:**
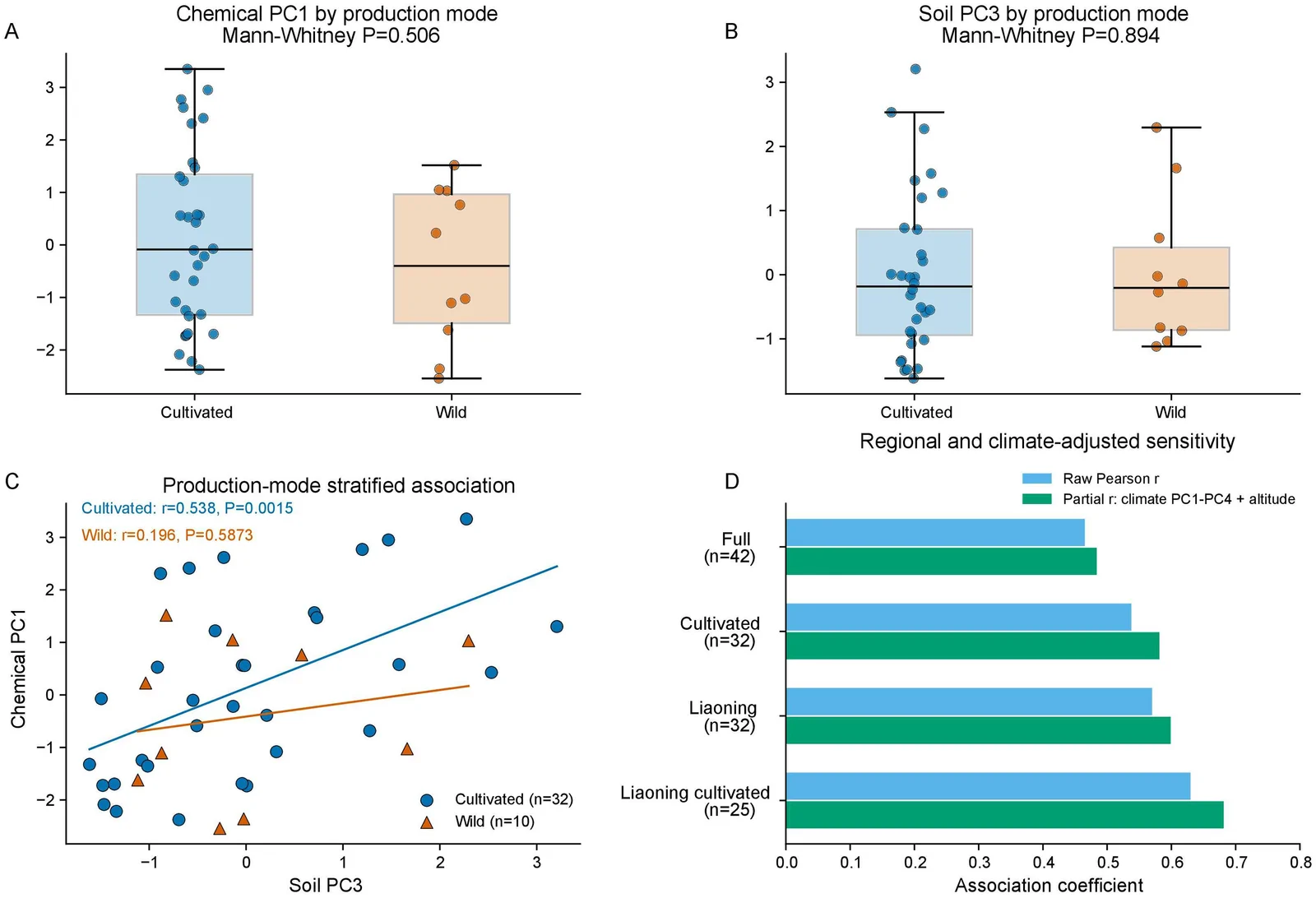
Production-mode, regional and climate-adjusted sensitivity analyses. **(A, B)** Chemical PC1 and soil PC3 in cultivated versus wild reference samples. **(C)** Production-mode-stratified soil PC3-chemical PC1 relationships; the wild subset is descriptive because n=10. **(D)** Raw Pearson coefficients and climate-adjusted partial correlations for the full dataset, cultivated samples, all Liaoning samples, and the 25 Liaoning cultivated samples. Climate adjustment used climate PC1-PC4 plus altitude.

Regional imbalance was addressed with two prespecified sensitivity analyses. The association remained significant among all 32 Liaoning samples (r=0.570, P = 6.61×10−4) and was stronger when analysis was restricted to the 25 Liaoning cultivated samples that constitute the central sampling frame of the study (r=0.630, P = 7.45×10−4). These results indicate that the full-data association is not generated solely by the small numbers of samples from other provinces.

The newly recovered climate matrix allowed a further confounding analysis. Nineteen BIOCLIM variables were reduced to four retained climate PCs explaining 98.17% of climatic variance, and both soil PC3 and chemical PC1 were residualized against these four climate PCs plus altitude. The full-data partial correlation remained significant (partial r=0.484, P = 0.0024, partial R²=0.234). Climate-adjusted partial correlations were also significant in cultivated samples (r=0.581, P = 0.0015), all Liaoning samples (r=0.599, P = 0.0010), and Liaoning cultivated samples (r=0.681, P = 0.00094) (**Figure 6D**; **Supplementary Table S5**).

## 4 Discussion

The revised analysis changes the emphasis of the ecological-quality interpretation. Rather than identifying a collection of single nutrients that independently “promote” particular compounds, the data support one reproducible multivariate pattern identified after exhaustive screening of all retained PCA axes: soil PC3 covaries with a chemical axis representing coordinated abundance of the seven targeted markers. The effect is moderate (r=0.465; R²=0.216), not deterministic, and remains after FDR correction, cultivated-sample restriction, Liaoning restriction and climate adjustment. At the same time, PCA axes describe combinations of field conditions and do not identify a causal nutrient treatment.

A multi-marker view is appropriate for Atractylodis Rhizoma. Pharmacopoeial and contemporary quality-control studies increasingly combine marker determination, chromatographic fingerprints and multivariate chemometrics because medicinal materials contain many active and co-varying constituents (Chinese Pharmacopoeia Commission, 2020; Zhang WJ. et al., 2021; Zhao et al., 2023; Ji et al., 2024; Xing et al., 2025; Zhang et al., 2025; Sun et al., 2026; Zhu et al., 2020). The seven markers measured here span major sesquiterpene, polyacetylene, lactone and processing-related chemical features of *A. chinensis*. Their positive loadings on chemical PC1 indicate that a substantial fraction of sample-to-sample variation reflects broad increases or decreases across compounds, whereas PC2 and later axes capture compound-specific contrasts. This architecture argues against ranking accessions solely by one compound or by a PCA derived from soil variables rather than from the chemical data themselves.

The observed association between soil PC3 and chemical PC1 is plausible in the context of plant nutrition, but several mechanisms could produce the same field pattern. In the recovered dataset, soil PC3 loaded most strongly on available K, alkali-hydrolyzed N and pH, with secondary contributions from Ca, Mn and organic matter. K affects osmotic regulation and enzyme activation, N influences growth and carbon-nitrogen balance, and pH modifies nutrient availability and rhizosphere processes. These factors also covary with management and microbial activity. Therefore, the PC3 relationship should be interpreted as an integrated field gradient rather than the direct biochemical action of one soil variable.

The contrast between nominal pairwise correlations and the FDR-corrected result is especially instructive. With 91 soil-marker tests, several P values below 0.05 are expected even when effect estimates are modest. The Benjamini-Hochberg procedure is less conservative than family-wise corrections but still guards against over-interpreting the largest values from a large correlation matrix (Benjamini and Hochberg, 1995). Here, none of the individual soil-marker pairs remained significant after correction. Consequently, recommendations such as “increase phosphorus, potassium, zinc, magnesium and copper to promote active-component accumulation” go beyond what this observational dataset can support. Those factors remain candidates for experimental manipulation, but they should be tested prospectively in replicated dose-response experiments.

Recent Atractylodes studies illustrate how such validation can be designed. Drought experiments in *A. lancea* and *A. chinensis* have combined physiological measurements with transcriptomics or metabolomics and shown that water stress can alter sesquiterpene accumulation and pathway expression (Zhang A. et al., 2021; Ma et al., 2024; Wu et al., 2026). Drip-irrigation work in *A. chinensis* has likewise connected controlled water regimes to yield and quality traits (Lv et al., 2026). Interspecific rhizosphere experiments in *A. lancea* demonstrated that root-exudate signaling can alter volatile-oil content and biosynthetic gene expression (Peng et al., 2025). Most directly, Duan et al. combined field environmental data, structural equation modeling and experimental validation in hairy roots and pot-grown *A. lancea* to test candidate mineral drivers (Duan et al., 2026). Such studies establish the evidentiary step needed to move from ecological association to agronomic recommendation.

Genetic background is an essential unresolved consideration. Tsusaka et al. found high broad-sense heritability for several major *A. lancea* essential-oil compounds, showing that provenance effects can persist even in common environments (Tsusaka et al., 2019). There are few formally registered *A. chinensis* cultivars, and cultivated materials are often derived from locally domesticated wild seed or rhizome sources. The current survey does not include molecular genotype or population-structure data, so variation attributed statistically to edaphic conditions may partly reflect correlated genetic differentiation, propagation history or genotype-by-environment interaction. Representative morphology photographs added as **Supplementary Figure S2** support species-level authentication but cannot resolve within-species genetic heterogeneity. For this reason, wild samples are described as reference materials rather than genetically matched controls.

The sampling structure also requires cautious interpretation. The 42 analytical samples were purposively selected rather than randomly drawn from the broader 69-site resource survey. Within Liaoning, 25 cultivated samples were selected to span the observed phytochemical range and seven wild references were added from corresponding distribution areas; other provinces were included to broaden production/distribution coverage. This design is appropriate for exploring covariation across a wide chemical and edaphic range but is not suitable for estimating region-level prevalence or mean differences. We therefore avoid treating province as a balanced factor. Importantly, the principal association remained significant in the 25 Liaoning cultivated samples, which are most closely aligned with the original objective of evaluating environmental variation in cultivated material.

Climate is undoubtedly relevant to *A. chinensis* ecology. During revision, the complete sample-level environmental workbook was recovered, including exact coordinates and 19 BIOCLIM variables. To limit collinearity, the BIOCLIM matrix was reduced to four retained climate PCs explaining 98.17% of climatic variance. Partial-correlation analysis controlling for these four climate PCs plus altitude did not attenuate the soil PC3-chemical PC1 relationship; the full-data partial coefficient was 0.484 (P = 0.0024). Thus, broad climatic structure does not explain the observed association. However, the recovered workbook does not provide direct soil-moisture dynamics, hydrological flow, irrigation history for every sample or rhizosphere microbiome data, so finer-scale water and biological confounding remains possible.

Analytically, the archived HPLC validation supports comparative quantification: all calibration curves had R² above 0.999, technical precision/stability/repeatability RSDs were low, and spike recoveries were close to 100%. However, LOD and LOQ cannot be reconstructed defensibly from the archived material because low-concentration replicate responses and baseline signal-to-noise measurements were not retained. We have therefore revised the terminology throughout the manuscript and describe the assay as validated for linearity, precision, stability, repeatability and recovery rather than as fully validated for quality-control deployment.

Several limitations define the scope of inference. First, the study is cross-sectional and observational and therefore cannot establish causality. Second, purposive selection of Liaoning cultivated samples to span phytochemical variation improves dynamic range but limits population-level representativeness. Third, molecular genotype is unavailable, and morphological authentication cannot partition genetic from environmental effects. Fourth, plant age, exact harvest timing, pre-harvest management and post-harvest handling were not retained uniformly at sample level and may contribute to residual variation. Fifth, the n=10 wild reference subset has limited power and is no longer presented as independent validation. Sixth, although broad climate and altitude can now be adjusted statistically, direct hydrological measurements and rhizosphere microbiome data are absent. The observed full-data effect explains approximately 21.6% of chemical PC1 variance, leaving substantial variation to other biological and environmental factors.

From an applied perspective, the analysis provides a focused experimental hypothesis. Instead of manipulating many micronutrients independently based on nominal correlations, future work can first reproduce the PC3-like nutrient state under controlled conditions. A factorial or response-surface experiment could vary organic-matter supply, available N and K within agronomically realistic ranges, use a common genetic background, measure both biomass and the seven compounds, and include rhizosphere microbial profiling. If a coordinated chemical-PC1 response is reproduced, individual components of the soil axis can then be separated mechanistically. This sequence would convert the present observational association into actionable ecological-cultivation evidence while minimizing unnecessary fertilizer recommendations.

Overall, the revised framework aligns the biological question with the statistical structure and sampling purpose of the dataset. Medicinal quality varies along multiple chemical dimensions, soils vary along correlated physicochemical dimensions, and the main analytical objective concerns cultivated materials. Exhaustive testing of all retained PC combinations, FDR correction, cultivated/Liaoning restriction and climate adjustment all support one moderate soil PC3-chemical PC1 association. The lack of a significant relationship in the small wild subset and the absence of genotype data appropriately constrain the conclusion to environmental covariation in the sampled materials rather than causal soil control of medicinal quality.

## 5 Conclusions

Across 42 *A. chinensis* rhizome-soil pairs from northern China, both targeted phytochemical profiles and soil environments were heterogeneous. The 42 analytical samples were purposively selected, with the main sampling frame consisting of 25 cultivated Liaoning samples and seven wild references, supplemented by ten samples from other production/distribution regions. After explicit correction for 91 pairwise tests, no individual soil-marker association was FDR-robust. Using an explicit Kaiser criterion, six soil PCs and two chemical PCs were retained, and exhaustive testing of all 12 cross-space combinations identified soil PC3-chemical PC1 as the only FDR-significant association (r=0.465, q=0.0229, R²=0.216). The relationship persisted in cultivated, Liaoning and Liaoning-cultivated subsets and after adjustment for four climate PCs plus altitude, but not in the small wild subset. The results therefore support a multivariate soil-phytochemistry association in cultivated *A. chinensis* while leaving genotype, direct hydrology, microbiome and other unmeasured factors as important sources of residual confounding.

## Data Availability

The original contributions presented in the study are included in the article/**Supplementary Material**. Further inquiries can be directed to the corresponding author.

## References

[B1] Applequist W. L. Brinckmann J. A. Cunningham A. B. Hart R. E. Heinrich M. Katerere D. R. . (2020). Scientists' warning on climate change and medicinal plants. Planta Med. 86, 10–18. doi: 10.1055/a-1041-3406 31731314

[B2] Bai J. Lu Y. Dong P. Cao Y. Liu J. W. Zhao C. Y. (2025). The impact of rhizosphere soil microorganisms on the medicinal active ingredients of atractylodes chinensis from different regions. Plant Signaling Behav. 20, 2473517. doi: 10.1080/15592324.2025.2473517 40105288 PMC12959219

[B3] Benjamini Y. Hochberg Y. (1995). Controlling the false discovery rate: a practical and powerful approach to multiple testing. J. R. Stat. Soc. Ser. B. 57, 289–300. doi: 10.1111/j.2517-6161.1995.tb02031.x40046247

[B4] Bulgarelli D. Schlaeppi K. Spaepen S. van Themaat E. V. L. Schulze-Lefert P. (2013). Structure and functions of the bacterial microbiota of plants. Annu. Rev. Plant Biol. 64, 807–838. doi: 10.1146/annurev-arplant-050312-120106 23373698

[B5] Chinese Pharmacopoeia Commission (2020). Pharmacopoeia of the People's Republic of China, Part I (Beijing: China Medical Science Press).

[B6] Duan W. Xiong F. Wang Y. Wang H. Peng Z. Wang Y. . (2026). Unveiling ecological drivers of medicinal quality in atractylodes lancea via structural equation modeling. Ind. Crops Prod. 250, 123863. doi: 10.1016/j.indcrop.2026.12386342574925

[B7] Efron B. Tibshirani R. J. (1993). An Introduction to the Bootstrap (New York: Chapman & Hall/CRC).

[B8] Fierer N. (2017). Embracing the unknown: disentangling the complexities of the soil microbiome. Nat. Rev. Microbiol. 15, 579–590. doi: 10.1038/nrmicro.2017.87 28824177

[B9] Guo L. P. Huang L. Q. Shao A. J. Lv D. M. Wu Z. G. (2005). Changes in soil nutrients in the rhizosphere of *Atractylodes*. Chin. J. Chin. Mater. Med. 30 (19), 1504–1507. 16335818

[B10] Isah T. (2019). Stress and defense responses in plant secondary metabolites production. Biol. Res. 52, 39. doi: 10.1186/s40659-019-0246-3 31358053 PMC6661828

[B11] Jangpangi D. Patni B. Chandola V. Chandra S. (2025). Medicinal plants in a changing climate: understanding the links between environmental stress and secondary metabolite synthesis. Front. Plant Sci. 16, 1587337. doi: 10.3389/fpls.2025.1587337 40584865 PMC12202592

[B12] Ji P. Yang X. Zhao X. (2024). Application of metabolomics in quality control of traditional Chinese medicines: a review. Front. Plant Sci. 15, 1463666. doi: 10.3389/fpls.2024.1463666 39619843 PMC11604444

[B13] Jolliffe I. T. Cadima J. (2016). Principal component analysis: a review and recent developments. Philos. Trans. R. Soc. A. 374, 20150202. doi: 10.1098/rsta.2015.0202 26953178 PMC4792409

[B14] Lu Y. Cao X. Qiao C. Li Y. Bai J. Zhang T. . (2026). Rhizosphere microbiome assemblage impacts soil chemical properties and medicinal quality in atractylodes chinensis. Front. Microbiol. 17, 1854479. doi: 10.3389/fmicb.2026.1854479 42516982 PMC13402554

[B15] Lv G. Wu Y. Li Z. Zhuang D. Guo Y. Wang Z. . (2026). Effects of drip irrigation regimes on leaf structure, photosynthesis, yield, and quality in atractylodes chinensis (DC.) koidz. Front. Agron. 7, 1711027. doi: 10.3389/fagro.2025.1711027

[B16] Ma S. Sun C. Su W. Zhao W. Zhang S. Su S. . (2024). Transcriptomic and physiological analysis of atractylodes chinensis in response to drought stress reveals the putative genes related to sesquiterpenoid biosynthesis. BMC Plant Biol. 24, 91. doi: 10.1186/s12870-024-04780-8 38317086 PMC10845750

[B17] Mendes R. Garbeva P. Raaijmakers J. M. (2013). The rhizosphere microbiome: significance of plant beneficial, plant pathogenic, and human pathogenic microorganisms. FEMS Microbiol. Rev. 37, 634–663. doi: 10.1111/1574-6976.12028 23790204

[B18] Pant P. Pandey S. Dall'Acqua S. (2021). The influence of environmental conditions on secondary metabolites in medicinal plants: a literature review. Chem. Biodivers. 18, e2100345. doi: 10.1002/cbdv.202100345 34533273

[B19] Peng Z. Cao M. Zhu S. Yang J. Yan B. Wan X. . (2025). Interspecific rhizosphere interactions enhance secondary metabolite synthesis and volatile oil content in atractylodes lancea through root exudates. Ind. Crops Prod. 236, 122084. doi: 10.1016/j.indcrop.2025.12208442574925

[B20] Ramakrishna A. Ravishankar G. A. (2011). Influence of abiotic stress signals on secondary metabolites in plants. Plant Signaling Behav. 6, 1720–1731. doi: 10.4161/psb.6.11.17613 22041989 PMC3329344

[B21] Sun J. Qiao J. Tang J. Cheng N. Gao M. Yang J. . (2026). Herbal textual research, phytochemistry, pharmacology and toxicity of atractylodis rhizoma: a comprehensive review. Molecules 31, 1015. doi: 10.3390/molecules31061015 41900114 PMC13029298

[B22] Sun J. Weng L. L. Xiao C. P. (2021a). Research progress on the synthesis and accumulation of volatile oil in medicinal plant *Atractylodes*. J. Chin. Med. Mater. 44, 1259–1265. doi: 10.13863/j.issn1001-4454.2021.05.042

[B23] Sun J. Weng L. L. Xiao C. P. Zhou X. L. Jiang Y. X. (2021b). Effects of drought stress on accumulation of three sesquiterpenes in *Atractylodes* and expression of key enzyme genes. J. Chin. Med. Mater. 44, 812–817. doi: 10.13863/j.issn1001-4454.2021.04.00

[B24] Tang J. Han Y. Pei L. Gu W. Qiu R. Wang S. . (2023). Comparative analysis of the rhizosphere microbiome and medicinally active ingredients of atractylodes lancea from different geographical origins. Open Life Sci. 18, 20220769. doi: 10.1515/biol-2022-0769 38027226 PMC10668115

[B25] Tsusaka T. Makino B. Ohsawa R. Ezura H. (2019). Genetic and environmental factors influencing the contents of essential oil compounds in atractylodes lancea. PloS One 14, e0217522. doi: 10.1371/journal.pone.0217522 31136627 PMC6538177

[B26] van der Heijden M. G. A. Bardgett R. D. van Straalen N. M. (2008). The unseen majority: soil microbes as drivers of plant diversity and productivity in terrestrial ecosystems. Ecol. Lett. 11, 296–310. doi: 10.1111/j.1461-0248.2007.01139.x 18047587

[B27] Wang M. Deng J. Duan G. Chen L. Huang X. Wang W. . (2023). Insights into the impacts of autotoxic allelochemicals from rhizosphere of atractylodes lancea on soil microenvironments. Front. Plant Sci. 14, 1136833. doi: 10.3389/fpls.2023.1136833 36968368 PMC10036400

[B28] Wang Y. Gu X. Zhao Y. Zhang X. Yang J. Zheng Y. . (2026). Assessing climate change impacts on habitat suitability and quality of atractylodes chinensis using ecological niche modeling. J. Nat. Conserv. 91, 127239. doi: 10.1016/j.jnc.2026.12723942574925

[B29] Wu Y. Lu Y. Ye Q. Jin B. Jiang B. Wang D. . (2026). Integrated transcriptome and metabolome analysis reveal the sesquiterpenoid biosynthesis mechanism of atractylodes chinensis under drought stress. Front. Plant Sci. 16, 1751860. doi: 10.3389/fpls.2025.1751860 41669635 PMC12884327

[B30] Xing Y. Liu M. Zhang J. Wu H. Ma G. Jiao L. . (2025). Four new polyynes and sesquiterpenoids from atractylodes chinensis. Fitoterapia 187, 106927. doi: 10.1016/j.fitote.2025.106927 41083077

[B31] You X. Wang C. Lu Z. Fu X. Tong Y. Song Y. . (2026). Integrating environmental variables and land use types to assess the habitat suitability and atractylodin content of two atractylodis rhizoma species under climate change. Front. Plant Sci. 17, 1866314. doi: 10.3389/fpls.2026.1866314 42582238 PMC13457460

[B32] Zhang A. Liu M. Gu W. Chen Z. Gu Y. Pei L. . (2021). Effect of drought on photosynthesis, total antioxidant capacity, bioactive component accumulation, and the transcriptome of atractylodes lancea. BMC Plant Biol. 21, 293. doi: 10.1186/s12870-021-03048-9 34171994 PMC8226357

[B33] Zhang C. Wang H. Lyu C. Wang Y. Sun J. Zhang Y. . (2023). Authenticating the geographic origins of atractylodes lancea rhizome chemotypes in China through metabolite marker identification. Front. Plant Sci. 14, 1237800. doi: 10.3389/fpls.2023.1237800 37841605 PMC10569125

[B34] Zhang S. Zhang L. Zou H. Qiu L. Zheng Y. Yang D. . (2021). Effects of light on secondary metabolite biosynthesis in medicinal plants. Front. Plant Sci. 12, 781236. doi: 10.3389/fpls.2021.781236 34956277 PMC8702564

[B35] Zhang W. J. Zhao Z. Y. Chang L. K. . (2021). Atractylodis rhizoma: a review of its traditional uses, phytochemistry, pharmacology, toxicology and quality control. J. Ethnopharmacol. 266, 113415. doi: 10.1016/j.jep.2020.113415 32987126 PMC7521906

[B36] Zhang Z. Tian Y. Qiao X. Li H. Ouyang L. Li X. . (2025). Integrated analysis of terpenoid profiles and full-length transcriptome reveals the central pathways of sesquiterpene biosynthesis in atractylodes chinensis. Int. J. Mol. Sci. 26, 1074. doi: 10.3390/ijms26031074 39940836 PMC11818032

[B37] Zhao Y. Chang X. Gu X. Li Y. Zheng Y. Fang H. (2023). Predictive analysis of quality markers of atractylodis rhizoma based on fingerprint and network pharmacology. J. AOAC Int. 106, 1402–1413. doi: 10.1093/jaoacint/qsad059 37208180

[B38] Zhu S. M. Ouyang L. M. Wang X. H. An R. Cui B. You L. S. (2020). Quality evaluation and grading standards of *Atractylodes chinensis* commercial medicinal materials. Chin. Tradit. Patent Med. 42 (6), 1536–1543. doi: 10.3969/j.issn.1001-1528.2020.06.028

