# Supplementary material for: Multivariate edaphic gradients are associated with phytochemical quality variation in Atractylodes chinensis across northern China
Source: Front Plant Sci. 2026 Oct 5;17:1962243. doi: 10.3389/fpls.2026.1962243 (PMC13678642; doi:10.3389/fpls.2026.1962243)
Supplement: Supplementary file 1 [file DataSheet1.docx]

**Supporting Information**

**Multivariate edaphic gradients are associated with phytochemical quality variation in *Atractylodes chinensis* across northern China**

# Figure S1. HPLC method-validation and representative-sample chromatograms

**Figure S1.** Archived HPLC chromatograms from the source thesis. (A) Linearity examination. (B) Precision. (C) Repeatability. (D) Stability. (E) Spike recovery. (F) Representative *A. chinensis* sample. Peak identities: 1, atractylon; 2, atractylodin; 3, β-eudesmol; 4, hinesol; 5, atractylenolide I; 6, atractylenolide III; 7, 5-HMF.

# **Figure S2. Representative morphology of cultivated and wild Atractylodes chinensis materials**

Figure S2. Representative field morphology used as supporting documentation for species-level authentication. (A,B) Cultivated *A. chinensis* plants. (C,D) Wild *A. chinensis* reference plants. These photographs do not establish genetic identity or exclude within-species genetic differentiation.

# Table S1. Restored metadata for the 42 analytical samples

| **Voucher** | **Original ID** | **Locality** | **Region** | **Original locality (Chinese)** | **Production mode** |
| --- | --- | --- | --- | --- | --- |
| LN1 | Z-LN1 | Liaoning Haicheng | Liaoning | 辽宁省鞍山市海城市1 | Cultivated |
| LN2 | Z-LN2 | Liaoning Haicheng | Liaoning | 辽宁省鞍山市海城市2 | Cultivated |
| LN3 | Z-LN3 | Liaoning Xiuyan | Liaoning | 辽宁省鞍山市岫岩县 | Cultivated |
| LN4 | Z-LN4 | Liaoning Fengcheng | Liaoning | 辽宁省丹东市凤城市1 | Cultivated |
| LN5 | Z-LN5 | Liaoning Fengcheng | Liaoning | 辽宁省丹东市凤城市2 | Cultivated |
| LN6 | Z-LN6 | Liaoning Fengcheng | Liaoning | 辽宁省丹东市凤城市3 | Cultivated |
| LN7 | Z-LN7 | Liaoning Kuandian | Liaoning | 辽宁省丹东市宽甸县1 | Cultivated |
| LN8 | Z-LN8 | Liaoning Kuandian | Liaoning | 辽宁省丹东市宽甸县2 | Cultivated |
| LN9 | Z-LN9 | Liaoning Benxi | Liaoning | 辽宁省本溪市本溪县 | Cultivated |
| LN10 | Z-LN10 | Liaoning Huanren | Liaoning | 辽宁省本溪市桓仁县1 | Cultivated |
| LN11 | Z-LN11 | Liaoning Huanren | Liaoning | 辽宁省本溪市桓仁县2 | Cultivated |
| LN12 | Z-LN12 | Liaoning Xinbin | Liaoning | 辽宁省抚顺市新宾县 | Cultivated |
| LN13 | Z-LN13 | Liaoning Qingyuan | Liaoning | 辽宁省抚顺市清原县1 | Cultivated |
| LN14 | Z-LN14 | Liaoning Qingyuan | Liaoning | 辽宁省抚顺市清原县2 | Cultivated |
| LN15 | Z-LN15 | Liaoning Fuxin | Liaoning | 辽宁省阜新市阜蒙县 | Cultivated |
| LN16 | Z-LN16 | Liaoning Kaiyuan | Liaoning | 辽宁省铁岭市开原市 | Cultivated |
| LN17 | Z-LN17 | Liaoning Chaoyang | Liaoning | 辽宁省朝阳市朝阳县1 | Cultivated |
| LN18 | Z-LN19 | Liaoning Kazuo | Liaoning | 辽宁省朝阳市喀左县 | Cultivated |
| LN19 | Z-LN23 | Liaoning Jianchang | Liaoning | 辽宁省葫芦岛市建昌县1 | Cultivated |
| LN20 | Z-LN24 | Liaoning Jianchang | Liaoning | 辽宁省葫芦岛市建昌县2 | Cultivated |
| LN21 | Z-LN25 | Liaoning Jianchang | Liaoning | 辽宁省葫芦岛市建昌县3 | Cultivated |
| LN22 | Z-LN30 | Liaoning Xingcheng | Liaoning | 辽宁省葫芦岛市兴城市 | Cultivated |
| LN23 | Z-LN31 | Liaoning Yixian | Liaoning | 辽宁省锦州市义县 | Cultivated |
| LN24 | Z-LN32 | Liaoning Liaoyang | Liaoning | 辽宁省辽阳市文圣区 | Cultivated |
| LN25 | Z-LN33 | Liaoning Dalian | Liaoning | 辽宁省大连市普兰店区 | Cultivated |
| LN26 | Y-LN5 | Liaoning Chaoyang | Liaoning | 辽宁省朝阳市朝阳县1 | Wild |
| LN27 | Y-LN14 | Liaoning Jianchang | Liaoning | 辽宁省葫芦岛市建昌县6 | Wild |
| LN28 | Y-LN19 | Liaoning Jianchang | Liaoning | 辽宁省葫芦岛市建昌县11 | Wild |
| LN29 | Y-LN21 | Liaoning Jinzhou | Liaoning | 辽宁省锦州市古塔区 | Wild |
| LN30 | Y-LN26 | Liaoning Linghai | Liaoning | 辽宁省锦州市凌海市1 | Wild |
| LN31 | Y-LN27 | Liaoning Linghai | Liaoning | 辽宁省锦州市凌海市2 | Wild |
| LN32 | Y-LN28 | Liaoning Lingyuan | Liaoning | 辽宁省朝阳市凌源市1 | Wild |
| HB1 | Z-HB3 | Hebei Longhua | Hebei | 河北省承德市隆化县1 | Cultivated |
| HB2 | Z-HB5 | Hebei Kuancheng | Hebei | 河北省承德市宽城县2 | Cultivated |
| HB3 | Y-HB4 | Hebei Kuancheng | Hebei | 河北省承德市宽城县1 | Wild |
| HB4 | Y-HB7 | Hebei Longhua | Hebei | 河北省承德市隆化县3 | Wild |
| JL1 | Z-JL1 | Jilin Tonghua | Jilin | 吉林省通化市通化县1 | Cultivated |
| JL2 | Z-JL2 | Jilin Tonghua | Jilin | 吉林省通化市通化县2 | Cultivated |
| JL3 | Z-JL4 | Jilin Tonghua | Jilin | 吉林省通化市通化县4 | Cultivated |
| HLJ | Z-HLJ1 | Heilongjiang Heihe | Heilongjiang | 黑龙江省黑河市嫩江镇 | Cultivated |
| NMG1 | Z-NM2 | Inner Mongolia Tongliao | Inner Mongolia | 内蒙古通辽市科尔沁区 | Cultivated |
| NMG2 | Y-NM1 | Inner Mongolia Tongliao | Inner Mongolia | 内蒙古自治区通辽市1 | Wild |

# Table S2. HPLC method-validation summary

| **Marker** | **Calibration_equation** | **R_squared** | **Linear_range_ug** | **Precision_RSD_pct** | **Stability_24h_RSD_pct** | **Repeatability_RSD_pct** | **Recovery_mean_pct** | **Recovery_RSD_pct** |
| --- | --- | --- | --- | --- | --- | --- | --- | --- |
| Atractylon | y =187.07x + 19.06 | 0.9992 | 1.20~11.9 | 2.222 | 1.207 | 2.461 | 102.637 | 2.752 |
| Atractylodin | y =1886.7x - 80.837 | 0.9992 | 1.05~10.5 | 1.315 | 2.372 | 1.323 | 96.986 | 2.626 |
| β-Eudesmol | y =378.22x+46.143 | 0.9991 | 0.94~9.4 | 2.946 | 2.922 | 2.791 | 96.079 | 2.793 |
| Hinesol | y =94.834x -2.2137 | 0.9994 | 1.26~12.6 | 1.692 | 2.21 | 2.828 | 95.512 | 3.857 |
| Atractylenolide I | y =340.44x + 3.1309 | 0.9996 | 0.93~9.3 | 1.385 | 1.677 | 2.414 | 99.802 | 3.543 |
| Atractylenolide III | y = 300.09x +0.4184 | 0.9994 | 0.58~5.8 | 1.345 | 2.298 | 0.944 | 103.053 | 1.388 |
| 5-HMF | y = 369.11x + 17.299 | 0.9991 | 0.40~4.0 | 2.746 | 2.924 | 1.282 | 102.644 | 1.56 |

# Table S3. Descriptive statistics for the seven phytochemical markers

| **Marker** | **mean** | **variance** | **std** | **min** | **max** | **CV_percent** |
| --- | --- | --- | --- | --- | --- | --- |
| Atractylon | 6.294857142857142 | 30.32181729616725 | 5.506524974624854 | 0.5 | 23.876 | 87.47656777045658 |
| Atractylodin | 3.6480952380952383 | 1.7848994053426246 | 1.336001274453967 | 0.961 | 6.519 | 36.62188586807637 |
| β-Eudesmol | 1.1971666666666667 | 1.2073876056910569 | 1.0988119064203195 | 0.133 | 4.672 | 91.78437196884194 |
| Hinesol | 2.7494285714285716 | 3.5434130313588863 | 1.8823955565605457 | 0.606 | 7.689 | 68.46497399939634 |
| Atractylenolide I | 1.661952380952381 | 3.3871319488966316 | 1.8404162433799132 | 0.395 | 11.055 | 110.73820552699974 |
| Atractylenolide III | 0.31338095238095237 | 0.061933412311266 | 0.24886424474252222 | 0.087 | 1.298 | 79.4126901624824 |
| 5-HMF | 0.21414285714285713 | 0.024673979094076648 | 0.15707953111107967 | 0.022 | 0.788 | 73.35268297381973 |

**Table S4. Descriptive statistics for the 13 soil variables**

| **Variable** | **mean** | **variance** | **std** | **min** | **max** | **CV_percent** |
| --- | --- | --- | --- | --- | --- | --- |
| Alkali-N | 124.92714285714287 | 4113.525806271777 | 64.13677421161573 | 31.85 | 346.85 | 51.33934287199511 |
| Organic matter | 14.997380952380954 | 67.04716126596979 | 8.188233097925938 | 2.65 | 34.58 | 54.59775359394329 |
| pH | 6.210714285714286 | 0.8494019163763064 | 0.9216300322669104 | 4.62 | 8.44 | 14.839356471232598 |
| Available P | 144.30714285714288 | 35635.18263066203 | 188.77283340211332 | 2.7 | 951.2 | 130.81322910605286 |
| Available K | 153.47619047619048 | 2779.279907084785 | 52.71887619330276 | 56.0 | 255.0 | 34.34987279116841 |
| Available B | 0.5650000000000001 | 0.16469878048780487 | 0.4058309752690212 | 0.07 | 1.86 | 71.82849119805684 |
| Ca | 12.245238095238097 | 223.0299475029036 | 14.934187205968177 | 0.49 | 80.74 | 121.9591410948208 |
| Mg | 8.412619047619048 | 17.548224680603948 | 4.189060119000914 | 2.24 | 23.4 | 49.79495797074643 |
| Fe | 30.823809523809526 | 120.57502903600464 | 10.980666147188186 | 3.3 | 59.1 | 35.623974832527715 |
| Mn | 767.547619047619 | 2194275.0342624853 | 1481.3085547118417 | 280.0 | 10087.0 | 192.9923978592839 |
| Zn | 63.476190476190474 | 528.3042973286875 | 22.984871053122912 | 38.0 | 158.0 | 36.21022446478479 |
| Cu | 15.571428571428571 | 80.8850174216028 | 8.99360981039331 | 5.0 | 51.0 | 57.757127222709336 |
| Mo | 1.130952380952381 | 0.2729210220673635 | 0.5224184358034883 | 0.2 | 2.9 | 46.192788007887394 |

# **Table S5. Sensitivity analyses for the soil PC3-chemical PC1 association**

| **Analysis** | **n** | **Coefficient/statistic** | **P value** | **Effect size** | **Interpretation** | **effect_label** |
| --- | --- | --- | --- | --- | --- | --- |
| Chemical PC1: cultivated vs wild | 32 vs 10 | Mann-Whitney U=183 | 0.506 | median difference=0.313 | No production-mode shift | median difference (cultivated-wild) |
| Soil PC3: cultivated vs wild | 32 vs 10 | Mann-Whitney U=155 | 0.894 | median difference=0.023 | No production-mode shift | median difference (cultivated-wild) |
| Soil PC3 vs chemical PC1: full | 42 | Pearson r=0.465 | 0.00191 | R²=0.216; Spearman ρ=0.459 | Significant | Pearson r; Spearman rho=0.5652, p=9.627e-05 |
| Cultivated | 32 | Pearson r=0.538 | 0.00151 | R²=0.289 | Significant | Pearson r; Spearman rho=0.5301, p=0.001807 |
| Wild reference | 10 | Pearson r=0.196 | 0.587 | R²=0.038 | Descriptive; underpowered | Pearson r; Spearman rho=0.7212, p=0.01857 |
| Liaoning | 32 | Pearson r=0.570 | 0.000661 | R²=0.325 | Significant | Pearson r; Spearman rho=0.6166, p=0.0001714 |
| Liaoning cultivated | 25 | Pearson r=0.630 | 0.000745 | R²=0.396 | Significant |  |
| Full, climate-adjusted | 42 | partial r=0.484 | 0.00241 | partial R²=0.234 | Climate PC1-PC4 + altitude |  |
| Cultivated, climate-adjusted | 32 | partial r=0.581 | 0.00147 | partial R²=0.338 | Climate PC1-PC4 + altitude |  |
| Liaoning, climate-adjusted | 32 | partial r=0.599 | 0.000966 | partial R²=0.359 | Climate PC1-PC4 + altitude |  |
| Liaoning cultivated, climate-adjusted | 25 | partial r=0.681 | 0.000943 | partial R²=0.464 | Climate PC1-PC4 + altitude |  |

# **Table S6. Chemical PCA loadings**

| **Marker** | **PC1** | **PC2** | **PC3** | **PC4** | **PC5** | **PC6** | **PC7** |
| --- | --- | --- | --- | --- | --- | --- | --- |
| Atractylon | 0.33586957110990695 | 0.032067872200746826 | 0.7680466211866014 | 0.3658733936149166 | 0.22061684635451287 | 0.21359059060397603 | -0.2609819859943354 |
| Atractylodin | 0.45847685261110693 | -0.2812436666441818 | 0.14944153505855948 | -0.21121203448372144 | -0.5534572760726891 | -0.5410364348783494 | -0.21147647476081718 |
| β-Eudesmol | 0.47577336410333565 | -0.11345921220591315 | -0.1144935054341677 | -0.40107341394687424 | 0.7236858563693886 | -0.20813872064827785 | 0.14055291271121245 |
| Hinesol | 0.4912776083483399 | -0.09089226999499957 | -0.2356025130415096 | -0.21082413879272988 | -0.2770044444444985 | 0.7572212286440705 | 0.017722691394903848 |
| Atractylenolide I | 0.3199292320435261 | 0.5938142491484553 | 0.08216650960511615 | 0.16307538367974905 | -0.18779728549073796 | -0.14978973454302225 | 0.6737805450717259 |
| Atractylenolide III | 0.16711105140007682 | 0.6732097742270173 | -0.30870548863105235 | 0.002107853250766791 | 0.04668059040219847 | -0.09095631205412635 | -0.6427338845946433 |
| 5-HMF | 0.28106192796533463 | -0.3048155038040015 | -0.4659440892784068 | 0.7678680952179412 | 0.08434258567117536 | -0.11934363963058664 | 0.003133564110290678 |

# **Table S7. Soil PCA loadings**

| **Variable** | **PC1** | **PC2** | **PC3** | **PC4** | **PC5** | **PC6** | **PC7** | **PC8** | **PC9** | **PC10** | **PC11** | **PC12** | **PC13** |
| --- | --- | --- | --- | --- | --- | --- | --- | --- | --- | --- | --- | --- | --- |
| Alkali-N | -0.10986894809930331 | 0.39694399197092994 | 0.5116685537766574 | -0.007277740670913955 | -0.2735362828069037 | -0.23849207898440733 | 0.029935983609432427 | 0.4934269053823779 | -0.05036153566714405 | -0.4188769871536707 | -0.08257641623833381 | 0.06289566230204978 | 0.060587290182661034 |
| Organic matter | -0.15556457642189198 | 0.448859026912317 | 0.18042339282680994 | 0.1773081156106692 | 0.1802711560475466 | -0.48260779541921833 | -0.3166378424655498 | -0.49047134702168754 | 0.1271501399954183 | 0.15983029596433918 | 0.12538794452908483 | -0.2061433423004316 | -0.06461217170094745 |
| pH | 0.2121611235890036 | -0.35954274746192305 | 0.47152613633057916 | -0.12893799678853618 | -0.29112090870973195 | 0.13503538708141177 | 0.04373893283985466 | -0.4472480670133723 | 0.10612952788281595 | -0.3362917112686483 | 0.06789509343173074 | -0.2850799075049001 | -0.267561226729872 |
| Available P | -0.2065407526461875 | 0.27086619449149774 | -0.06254732123843604 | 0.22742971809309806 | 0.42213117780019327 | 0.5706390593780251 | -0.10543568011416554 | -0.09083860962683707 | 0.17973766874320288 | -0.49890097075961287 | -0.12243132121449787 | -0.023921453042913862 | -0.09151230842353253 |
| Available K | -0.1856702650801634 | 0.08319464192589902 | 0.5478462488872045 | 0.21941054029869064 | 0.006097569687071689 | 0.39163489714051264 | 0.275547644677583 | -0.020623916169737865 | 0.055979516419572446 | 0.5868913876685946 | -0.06464685177283612 | 0.16563031902601863 | -0.0370669392196319 |
| Available B | 0.06967637030406128 | 0.42405935928664223 | -0.05732803744040206 | -0.41156682425631597 | -0.30899640078110896 | 0.4012496593602359 | -0.28941219707692817 | -0.18460031183790584 | -0.4683413168625143 | 0.1181276484724132 | 0.009197651859564499 | -0.1003634135787788 | 0.1577124178321848 |
| Ca | 0.39431695015355 | -0.04044830174375533 | 0.25098821234016294 | -0.2587350468975474 | 0.3040019660824644 | -0.03512013062776757 | -0.11510998253473478 | -0.12669112221183568 | 0.2625714467943693 | -0.015208713684404988 | -0.33260800596475176 | 0.19438115084084373 | 0.6102461928758128 |
| Mg | 0.3897909364234074 | -0.0019398145807422943 | 0.04983948238798724 | 0.3895103867776606 | -0.04634256559384689 | -0.03185978756595574 | -0.233548269398862 | -0.16075276281479534 | -0.32297698057147733 | -0.10880777894240717 | 0.04723727148339692 | 0.6678476988851085 | -0.2142495082538829 |
| Fe | 0.33528746823632216 | 0.061914400180875385 | -0.057340409139208334 | 0.519504578647735 | -0.02662257828594871 | -0.03198285720901504 | 0.21485946955170013 | 0.000567984874496519 | -0.33145542982590936 | 0.013648550217810154 | -0.42162358540704825 | -0.5005403436788514 | 0.15595942914503408 |
| Mn | 0.2930534555435121 | -0.04226129665482306 | 0.2440848696294896 | -0.24683529722632264 | 0.5718780181702752 | -0.004221806057063289 | -0.18159115407953375 | 0.3482686908340541 | -0.26490194441790477 | 0.1322912068028111 | 0.0960620621219867 | -0.22793838790889748 | -0.4020472806485492 |
| Zn | 0.36225865428110526 | 0.32660520547997096 | -0.20392230606309655 | -0.12076522401031664 | -0.24214107595972464 | 0.028433161286392842 | -0.006563126090091575 | 0.0921300059009028 | 0.5049272697642228 | 0.16719061644269462 | -0.37210377942575823 | 0.028323658694139095 | -0.46453170041411973 |
| Cu | 0.40508336478524853 | 0.13989663084324322 | -0.013120098185840433 | 0.25992062253559717 | -0.10388848303121882 | 0.19547430997461065 | -0.07121449558972766 | 0.21044939825242429 | 0.30372338069513083 | 0.045223060551169515 | 0.6803023271670464 | -0.17169384280397604 | 0.25230736749369376 |
| Mo | 0.1889023492191012 | 0.3436169441046063 | -0.07662090273718324 | -0.22586177294066404 | 0.20609455546544073 | -0.09828897930991441 | 0.7576800065279758 | -0.23921174148955002 | -0.12147872048546593 | -0.13729857499673748 | 0.22906270422354919 | 0.13273251600360822 | -0.04718511066854046 |

# **Table S8. Raw phytochemical concentrations for all 42 analytical samples**

| **Voucher** | **Atractylon** | **Atractylodin** | **β-Eudesmol** | **Hinesol** | **Atractylenolide I** | **Atractylenolide III** | **5-HMF** |
| --- | --- | --- | --- | --- | --- | --- | --- |
| LN1 | 1.34 | 3.517 | 0.133 | 1.035 | 0.857 | 0.166 | 0.246 |
| LN2 | 1.424 | 3.029 | 0.248 | 0.663 | 0.823 | 0.373 | 0.124 |
| LN3 | 0.5 | 3.517 | 0.184 | 0.933 | 0.865 | 0.202 | 0.093 |
| LN4 | 1.884 | 4.524 | 0.741 | 3.06 | 2.591 | 1.1 | 0.046 |
| LN5 | 5.133 | 5.294 | 2.959 | 7.216 | 2.0 | 0.138 | 0.572 |
| LN6 | 1.892 | 3.629 | 0.354 | 4.735 | 0.677 | 0.112 | 0.788 |
| LN7 | 6.542 | 4.005 | 1.229 | 2.742 | 1.059 | 0.273 | 0.251 |
| LN8 | 0.903 | 4.782 | 0.363 | 0.962 | 1.28 | 0.221 | 0.164 |
| LN9 | 5.35 | 6.519 | 4.672 | 3.539 | 1.143 | 0.261 | 0.3 |
| LN10 | 7.341 | 1.679 | 0.405 | 0.606 | 0.963 | 0.39 | 0.271 |
| LN11 | 5.246 | 3.061 | 1.299 | 2.058 | 0.486 | 0.229 | 0.225 |
| LN12 | 0.763 | 4.848 | 1.867 | 3.744 | 0.395 | 0.341 | 0.341 |
| LN13 | 7.686 | 3.748 | 1.819 | 1.858 | 2.252 | 0.396 | 0.422 |
| LN14 | 6.154 | 3.546 | 0.372 | 3.141 | 0.893 | 0.212 | 0.311 |
| LN15 | 9.755 | 3.469 | 1.764 | 2.824 | 0.761 | 0.131 | 0.274 |
| LN16 | 16.988 | 3.705 | 0.371 | 1.081 | 1.066 | 0.252 | 0.127 |
| LN17 | 6.387 | 2.452 | 1.577 | 2.271 | 0.555 | 0.137 | 0.093 |
| LN18 | 6.818 | 3.065 | 1.707 | 3.033 | 11.055 | 1.298 | 0.438 |
| LN19 | 9.4 | 5.82 | 3.283 | 7.689 | 4.571 | 0.457 | 0.108 |
| LN20 | 1.014 | 0.961 | 1.698 | 1.425 | 3.107 | 0.788 | 0.034 |
| LN21 | 6.579 | 2.398 | 0.429 | 0.895 | 0.539 | 0.121 | 0.199 |
| LN22 | 2.27 | 2.164 | 0.531 | 0.798 | 2.234 | 0.501 | 0.21 |
| LN23 | 1.877 | 2.833 | 0.524 | 6.908 | 1.262 | 0.461 | 0.071 |
| LN24 | 0.84 | 2.755 | 0.464 | 2.024 | 0.807 | 0.126 | 0.142 |
| LN25 | 2.592 | 2.419 | 0.311 | 1.178 | 0.518 | 0.138 | 0.022 |
| LN26 | 4.622 | 1.77 | 0.18 | 3.39 | 1.204 | 0.499 | 0.111 |
| LN27 | 2.0 | 2.41 | 0.421 | 2.067 | 0.974 | 0.192 | 0.088 |
| LN28 | 1.038 | 2.149 | 0.35 | 1.264 | 0.561 | 0.089 | 0.187 |
| LN29 | 23.876 | 5.556 | 1.111 | 1.254 | 2.275 | 0.112 | 0.219 |
| LN30 | 16.606 | 3.744 | 0.607 | 3.575 | 1.404 | 0.482 | 0.298 |
| LN31 | 18.152 | 3.004 | 0.194 | 2.149 | 5.814 | 0.351 | 0.028 |
| LN32 | 4.905 | 5.34 | 1.557 | 3.572 | 1.992 | 0.249 | 0.316 |
| HB1 | 3.976 | 2.984 | 1.244 | 2.602 | 1.478 | 0.261 | 0.103 |
| HB2 | 4.598 | 3.658 | 0.802 | 1.168 | 0.732 | 0.173 | 0.085 |
| HB3 | 2.316 | 1.797 | 0.538 | 0.715 | 0.608 | 0.087 | 0.142 |
| HB4 | 4.689 | 2.506 | 0.647 | 1.801 | 0.857 | 0.159 | 0.218 |
| JL1 | 5.755 | 5.59 | 3.317 | 6.808 | 1.311 | 0.176 | 0.201 |
| JL2 | 7.87 | 4.952 | 2.909 | 5.763 | 1.058 | 0.322 | 0.523 |
| JL3 | 9.665 | 5.249 | 2.552 | 3.194 | 1.432 | 0.243 | 0.154 |
| HLJ | 7.548 | 5.198 | 3.214 | 2.701 | 1.047 | 0.333 | 0.083 |
| NMG1 | 17.354 | 5.505 | 0.519 | 4.398 | 2.872 | 0.186 | 0.136 |
| NMG2 | 12.736 | 4.069 | 0.815 | 2.637 | 1.424 | 0.424 | 0.23 |

Values are mg g−1 dry weight. These are the complete sample-level values used for the revised chemical PCA and descriptive statistics.

# **Table S9. Raw soil physicochemical data for all 42 analytical samples**

| **Voucher** | **Alkali-N** | **Organic matter** | **pH** | **Available P** | **Available K** | **Available B** | **Ca** | **Mg** | **Fe** | **Mn** | **Zn** | **Cu** | **Mo** |
| --- | --- | --- | --- | --- | --- | --- | --- | --- | --- | --- | --- | --- | --- |
| LN1 | 103.6 | 34.12 | 5.56 | 154.6 | 181.0 | 0.2 | 9.17 | 9.41 | 39.4 | 532.0 | 74.0 | 18.0 | 1.3 |
| LN2 | 52.5 | 11.9 | 5.94 | 73.2 | 87.0 | 0.69 | 7.22 | 8.58 | 22.6 | 352.0 | 52.0 | 10.0 | 1.0 |
| LN3 | 56.35 | 8.51 | 5.82 | 409.0 | 114.0 | 0.42 | 8.91 | 6.18 | 22.6 | 346.0 | 55.0 | 5.0 | 0.7 |
| LN4 | 115.5 | 15.34 | 4.83 | 62.6 | 211.0 | 0.11 | 4.07 | 6.7 | 21.7 | 335.0 | 40.0 | 5.0 | 1.1 |
| LN5 | 166.25 | 15.26 | 7.29 | 196.4 | 234.0 | 1.86 | 7.32 | 6.56 | 20.5 | 456.0 | 49.0 | 16.0 | 1.8 |
| LN6 | 260.4 | 33.88 | 6.27 | 433.9 | 246.0 | 0.07 | 3.72 | 7.81 | 29.8 | 418.0 | 38.0 | 8.0 | 1.1 |
| LN7 | 81.55 | 16.73 | 5.28 | 625.9 | 159.0 | 0.65 | 5.5 | 12.9 | 41.6 | 709.0 | 54.0 | 14.0 | 0.9 |
| LN8 | 31.85 | 6.22 | 7.25 | 69.9 | 93.0 | 0.36 | 15.3 | 23.4 | 59.1 | 618.0 | 73.0 | 36.0 | 1.3 |
| LN9 | 130.55 | 17.73 | 6.01 | 82.1 | 90.0 | 0.24 | 10.5 | 8.32 | 43.1 | 701.0 | 51.0 | 15.0 | 1.1 |
| LN10 | 62.65 | 9.6 | 5.71 | 194.3 | 128.0 | 0.22 | 0.49 | 2.24 | 22.1 | 529.0 | 54.0 | 8.0 | 1.6 |
| LN11 | 107.1 | 23.54 | 5.88 | 90.7 | 185.0 | 0.69 | 2.97 | 5.94 | 32.6 | 602.0 | 44.0 | 11.0 | 0.8 |
| LN12 | 95.2 | 8.25 | 6.39 | 384.3 | 211.0 | 0.39 | 10.7 | 7.18 | 37.0 | 667.0 | 81.0 | 31.0 | 1.1 |
| LN13 | 128.45 | 19.43 | 5.02 | 951.2 | 215.0 | 1.19 | 6.41 | 3.26 | 25.0 | 280.0 | 56.0 | 10.0 | 1.0 |
| LN14 | 106.05 | 23.41 | 5.45 | 132.7 | 56.0 | 0.47 | 3.38 | 5.85 | 24.2 | 465.0 | 58.0 | 15.0 | 1.0 |
| LN15 | 90.65 | 15.26 | 7.81 | 57.2 | 219.0 | 0.27 | 15.8 | 6.84 | 25.8 | 440.0 | 41.0 | 8.0 | 0.9 |
| LN16 | 127.75 | 20.7 | 4.62 | 401.6 | 173.0 | 0.44 | 4.03 | 5.69 | 3.3 | 420.0 | 52.0 | 12.0 | 1.1 |
| LN17 | 45.5 | 8.98 | 7.76 | 49.5 | 223.0 | 0.32 | 25.9 | 5.95 | 22.7 | 394.0 | 41.0 | 14.0 | 0.7 |
| LN18 | 204.05 | 11.4 | 7.8 | 43.2 | 126.0 | 0.32 | 10.4 | 10.6 | 29.2 | 546.0 | 56.0 | 16.0 | 1.1 |
| LN19 | 239.05 | 13.52 | 7.56 | 192.8 | 223.0 | 1.06 | 10.9 | 7.71 | 22.0 | 487.0 | 54.0 | 16.0 | 1.2 |
| LN20 | 73.85 | 13.43 | 5.75 | 113.4 | 121.0 | 0.19 | 13.0 | 11.8 | 52.6 | 616.0 | 46.0 | 11.0 | 2.0 |
| LN21 | 122.24 | 9.8 | 5.4 | 100.7 | 92.0 | 1.14 | 2.73 | 7.25 | 29.3 | 560.0 | 76.0 | 16.0 | 0.4 |
| LN22 | 45.85 | 9.28 | 5.53 | 196.3 | 112.0 | 0.29 | 2.4 | 6.02 | 27.6 | 505.0 | 47.0 | 14.0 | 1.2 |
| LN23 | 59.85 | 8.81 | 6.07 | 118.2 | 158.0 | 0.48 | 6.71 | 7.08 | 28.2 | 449.0 | 46.0 | 13.0 | 0.4 |
| LN24 | 63.35 | 11.17 | 6.42 | 85.1 | 223.0 | 0.31 | 4.46 | 9.34 | 41.9 | 518.0 | 71.0 | 23.0 | 1.5 |
| LN25 | 173.6 | 21.39 | 4.92 | 186.8 | 108.0 | 0.56 | 2.9 | 2.73 | 16.0 | 303.0 | 46.0 | 18.0 | 1.1 |
| LN26 | 147.39 | 17.9 | 5.57 | 13.2 | 131.0 | 1.34 | 9.1 | 7.93 | 29.6 | 764.0 | 100.0 | 17.0 | 1.8 |
| LN27 | 107.04 | 2.65 | 6.21 | 5.2 | 133.0 | 1.19 | 6.74 | 8.2 | 33.7 | 471.0 | 82.0 | 14.0 | 1.9 |
| LN28 | 171.11 | 4.05 | 5.8 | 9.3 | 176.0 | 1.02 | 7.45 | 6.35 | 20.7 | 587.0 | 77.0 | 7.0 | 0.9 |
| LN29 | 87.84 | 9.52 | 7.4 | 5.6 | 114.0 | 0.69 | 80.74 | 15.71 | 38.3 | 10087.0 | 81.0 | 31.0 | 1.8 |
| LN30 | 193.35 | 22.73 | 6.24 | 8.5 | 95.0 | 0.92 | 11.75 | 7.95 | 30.8 | 469.0 | 81.0 | 14.0 | 1.4 |
| LN31 | 97.72 | 25.91 | 5.66 | 5.5 | 114.0 | 1.13 | 10.71 | 7.57 | 30.0 | 560.0 | 101.0 | 16.0 | 0.6 |
| LN32 | 126.07 | 11.96 | 6.95 | 4.5 | 73.0 | 0.91 | 60.92 | 11.56 | 43.2 | 979.0 | 158.0 | 32.0 | 2.9 |
| HB1 | 104.65 | 6.25 | 7.72 | 43.4 | 94.0 | 0.41 | 6.15 | 4.06 | 19.1 | 434.0 | 55.0 | 10.0 | 0.6 |
| HB2 | 179.52 | 13.76 | 6.32 | 97.0 | 161.0 | 0.7 | 19.75 | 21.11 | 55.9 | 852.0 | 112.0 | 51.0 | 0.5 |
| HB3 | 104.83 | 3.67 | 6.13 | 20.1 | 155.0 | 0.32 | 13.08 | 9.46 | 37.6 | 777.0 | 65.0 | 12.0 | 0.4 |
| HB4 | 124.63 | 4.46 | 8.44 | 2.7 | 175.0 | 0.23 | 27.76 | 9.41 | 26.7 | 565.0 | 61.0 | 16.0 | 0.2 |
| JL1 | 65.45 | 7.37 | 5.83 | 77.6 | 163.0 | 0.21 | 9.43 | 8.74 | 44.5 | 763.0 | 67.0 | 19.0 | 1.5 |
| JL2 | 203.35 | 13.57 | 5.31 | 71.8 | 135.0 | 0.16 | 2.12 | 5.22 | 25.7 | 317.0 | 56.0 | 10.0 | 1.6 |
| JL3 | 184.1 | 24.28 | 5.54 | 185.2 | 210.0 | 0.34 | 3.97 | 6.79 | 34.0 | 620.0 | 64.0 | 15.0 | 1.4 |
| HLJ | 346.85 | 22.74 | 6.27 | 85.9 | 255.0 | 0.39 | 6.77 | 6.49 | 37.3 | 765.0 | 39.0 | 8.0 | 1.0 |
| NMG1 | 130.1 | 16.83 | 6.74 | 15.3 | 163.0 | 0.07 | 16.1 | 6.83 | 22.9 | 471.0 | 53.0 | 11.0 | 1.0 |
| NMG2 | 129.2 | 34.58 | 6.38 | 4.5 | 111.0 | 0.76 | 26.87 | 14.61 | 24.7 | 508.0 | 59.0 | 8.0 | 0.6 |

The 13-variable matrix is the complete sample-level soil dataset used for PCA and soil-marker association testing. Units follow the column headings in Materials and Methods.

# **Table S10. PCA eigenvalues, explained variance and component-retention criteria**

| **Dataset** | **PC** | **Eigenvalue** | **Explained_variance_pct** | **Cumulative_pct** | **Kaiser_retained** |
| --- | --- | --- | --- | --- | --- |
| Chemical | 1 | 2.702221896269777 | 37.68404685274179 | 37.68404685274179 | Yes |
| Chemical | 2 | 1.664838982666755 | 23.21714227528468 | 60.901189128026466 | Yes |
| Chemical | 3 | 0.9502129082445512 | 13.251268448308368 | 74.15245757633484 | No |
| Chemical | 4 | 0.7476545030380058 | 10.42647436209464 | 84.57893193842948 | No |
| Chemical | 5 | 0.45030517209832244 | 6.279766005452797 | 90.85869794388228 | No |
| Chemical | 6 | 0.41480254817466833 | 5.784661386109321 | 96.64335932999161 | No |
| Chemical | 7 | 0.2406956968249927 | 3.3566406700084017 | 100.0 | No |
| Soil | 1 | 4.00929947507911 | 30.106461259751544 | 30.106461259751544 | Yes |
| Soil | 2 | 1.6598316616721815 | 12.463937386183042 | 42.57039864593459 | Yes |
| Soil | 3 | 1.491648194351846 | 11.201021239638399 | 53.77141988557299 | Yes |
| Soil | 4 | 1.4213044340062084 | 10.67279886341658 | 64.44421874898957 | Yes |
| Soil | 5 | 1.092607491098657 | 8.204561746345222 | 72.64878049533479 | Yes |
| Soil | 6 | 1.0203841621577519 | 7.662225393492273 | 80.31100588882705 | Yes |
| Soil | 7 | 0.8563697785914304 | 6.43061555352539 | 86.74162144235244 | No |
| Soil | 8 | 0.4669508520364838 | 3.5064074969772574 | 90.2480289393297 | No |
| Soil | 9 | 0.43693246880207004 | 3.280994729099792 | 93.5290236684295 | No |
| Soil | 10 | 0.3757062210974028 | 2.821237191390752 | 96.35026085982025 | No |
| Soil | 11 | 0.24064054519073846 | 1.8070077569267897 | 98.15726861674705 | No |
| Soil | 12 | 0.17967927632132424 | 1.3492399870282583 | 99.50650860377532 | No |
| Soil | 13 | 0.0657186103265091 | 0.49349139622470173 | 100.00000000000003 | No |
| Climate | 1 | 7.941985598924711 | 40.804687914274794 | 40.804687914274794 | Yes |
| Climate | 2 | 7.3092016382807525 | 37.55354225181837 | 78.35823016609316 | Yes |
| Climate | 3 | 2.520448483394081 | 12.94967265904226 | 91.30790282513543 | Yes |
| Climate | 4 | 1.335137406519964 | 6.859728529739158 | 98.1676313548746 | Yes |
| Climate | 5 | 0.2266975183361923 | 1.1647366230305611 | 99.33236797790515 | No |
| Climate | 6 | 0.06954143229042736 | 0.3572930731713684 | 99.68966105107653 | No |
| Climate | 7 | 0.025189011657498196 | 0.1294172278142137 | 99.81907827889074 | No |
| Climate | 8 | 0.018594498450427925 | 0.09553564366761207 | 99.91461392255835 | No |

The Kaiser criterion (eigenvalue >1) retained chemical PC1-PC2, soil PC1-PC6 and climate PC1-PC4. The retained climate PCs explained 98.17% of the variance among the 19 BIOCLIM variables.

# **Table S11. Complete cross-space associations among retained soil and chemical PCs**

| **Soil_PC** | **Chemical_PC** | **Pearson_r** | **Pearson_P** | **Pearson_q_BH** | **Spearman_rho** | **Spearman_P** | **Spearman_q_BH** |
| --- | --- | --- | --- | --- | --- | --- | --- |
| 1 | 1 | 0.0188 | 0.9057 | 0.9057 | -0.0161 | 0.9193 | 0.9833 |
| 1 | 2 | -0.0230 | 0.8852 | 0.9057 | 0.0169 | 0.9152 | 0.9833 |
| 2 | 1 | 0.2489 | 0.1119 | 0.6715 | 0.2548 | 0.1034 | 0.4135 |
| 2 | 2 | -0.0851 | 0.5919 | 0.9057 | -0.0686 | 0.6658 | 0.9833 |
| 3 | 1 | 0.4651 | 0.0019 | 0.0229 | 0.4590 | 0.0022 | 0.0268 |
| 3 | 2 | -0.1802 | 0.2534 | 0.8371 | -0.2659 | 0.0888 | 0.4135 |
| 4 | 1 | -0.0757 | 0.6336 | 0.9057 | 0.0075 | 0.9622 | 0.9833 |
| 4 | 2 | -0.1018 | 0.5212 | 0.9057 | -0.1792 | 0.2563 | 0.7688 |
| 5 | 1 | 0.0249 | 0.8755 | 0.9057 | -0.0082 | 0.9590 | 0.9833 |
| 5 | 2 | -0.0578 | 0.7163 | 0.9057 | -0.0033 | 0.9833 | 0.9833 |
| 6 | 1 | -0.0503 | 0.7519 | 0.9057 | -0.1536 | 0.3316 | 0.7959 |
| 6 | 2 | -0.1710 | 0.2790 | 0.8371 | -0.1242 | 0.4332 | 0.8663 |

All six retained soil PCs were tested against both retained chemical PCs (12 tests). BH-FDR correction was applied across the complete set. Soil PC3-chemical PC1 was the only FDR-significant pair for both Pearson and Spearman analyses.

# **Table S12. Exact geographic coordinates and altitude of the 42 analytical samples**

| **Voucher** | **Latitude** | **Longitude** | **Altitude** |
| --- | --- | --- | --- |
| LN1 | 40.7075 | 122.9975 | 144 |
| LN2 | 40.79994 | 122.987968 | 130.21 |
| LN3 | 40.288116 | 123.587498 | 57.87 |
| LN4 | 40.319336 | 123.841614 | 64 |
| LN5 | 40.743946 | 124.250086 | 262 |
| LN6 | 40.45918 | 124.072422 | 368 |
| LN7 | 40.677603 | 124.777761 | 249.6 |
| LN8 | 40.527868 | 124.541171 | 221 |
| LN9 | 41.392655 | 125.03539 | 280.62 |
| LN10 | 41.391475 | 125.086442 | 387.34 |
| LN11 | 41.396667 | 125.031389 | 400.28 |
| LN12 | 41.709028 | 124.826632 | 213.52 |
| LN13 | 42.357373 | 124.959187 | 262 |
| LN14 | 42.113035 | 125.078209 | 409.71 |
| LN15 | 42.32387 | 121.914223 | 211.3 |
| LN16 | 42.418637 | 124.801161 | 307.85 |
| LN17 | 41.366978 | 120.097518 | 272 |
| LN18 | 41.167306 | 119.78185 | 326.35 |
| LN19 | 40.564513 | 119.253968 | 557.2 |
| LN20 | 40.76269 | 120.126135 | 141.5 |
| LN21 | 40.783758 | 119.737292 | 547.17 |
| LN22 | 40.78463 | 120.364109 | 201.54 |
| LN23 | 41.389511 | 121.266472 | 47.82 |
| LN24 | 41.260556 | 123.1375 | 21 |
| LN25 | 39.644203 | 122.424022 | 59.65 |
| LN26 | 41.708017 | 120.299596 | 508.72 |
| LN27 | 40.800901 | 118.931903 | 779.83 |
| LN28 | 40.673832 | 119.810122 | 427.96 |
| LN29 | 40.752496 | 120.404911 | 245.71 |
| LN30 | 41.583099 | 121.678689 | 120.35 |
| LN31 | 41.387402 | 121.603942 | 174.33 |
| LN32 | 41.272639 | 123.290073 | 140.48 |
| HB1 | 41.321553 | 117.697637 | 546.23 |
| HB2 | 40.550616 | 118.755495 | 683.67 |
| HB3 | 40.546321 | 118.750041 | 806.85 |
| HB4 | 41.319344 | 117.693867 | 613.92 |
| JL1 | 41.740003 | 125.572878 | 476 |
| JL2 | 41.553621 | 125.553691 | 418 |
| JL3 | 41.711107 | 125.700474 | 539 |
| HLJ | 49.166622 | 125.345461 | 345 |
| NMG1 | 43.678586 | 122.492634 | 177.8 |
| NMG2 | 45.147833 | 119.814202 | 970.05 |

# **Table S13. Sample-level bioclimatic variables used in climate sensitivity analysis**

| **Voucher** | **altitude** | **bio1** | **bio2** | **bio3** | **bio4** | **bio5** | **bio6** | **bio7** | **bio8** | **bio9** | **bio10** | **bio11** | **bio12** | **bio13** | **bio14** | **bio15** | **bio16** | **bio17** | **bio18** | **bio19** |
| --- | --- | --- | --- | --- | --- | --- | --- | --- | --- | --- | --- | --- | --- | --- | --- | --- | --- | --- | --- | --- |
| LN1 | 105.0 | 82.0 | 101.0 | 23.0 | 11802.0 | 278.0 | -153.0 | 431.0 | 226.0 | -79.0 | 226.0 | -79.0 | 760.0 | 202.0 | 8.0 | 99.0 | 470.0 | 28.0 | 470.0 | 28.0 |
| LN2 | 105.0 | 83.0 | 102.0 | 23.0 | 11827.0 | 279.0 | -154.0 | 433.0 | 227.0 | -79.0 | 227.0 | -79.0 | 753.0 | 200.0 | 8.0 | 99.0 | 466.0 | 28.0 | 466.0 | 28.0 |
| LN3 | 39.0 | 85.0 | 98.0 | 23.0 | 11331.0 | 278.0 | -141.0 | 419.0 | 223.0 | -70.0 | 223.0 | -70.0 | 848.0 | 232.0 | 10.0 | 101.0 | 529.0 | 32.0 | 529.0 | 32.0 |
| LN4 | 83.0 | 81.0 | 100.0 | 23.0 | 11338.0 | 276.0 | -146.0 | 422.0 | 219.0 | -74.0 | 219.0 | -74.0 | 906.0 | 252.0 | 10.0 | 102.0 | 570.0 | 33.0 | 570.0 | 33.0 |
| LN5 | 264.0 | 67.0 | 115.0 | 25.0 | 11751.0 | 270.0 | -180.0 | 450.0 | 210.0 | -94.0 | 210.0 | -94.0 | 1010.0 | 285.0 | 10.0 | 106.0 | 654.0 | 35.0 | 654.0 | 35.0 |
| LN6 | 69.0 | 80.0 | 106.0 | 24.0 | 11467.0 | 278.0 | -152.0 | 430.0 | 220.0 | -76.0 | 220.0 | -76.0 | 961.0 | 273.0 | 10.0 | 104.0 | 614.0 | 33.0 | 614.0 | 33.0 |
| LN7 | 230.0 | 73.0 | 115.0 | 25.0 | 11761.0 | 272.0 | -182.0 | 454.0 | 216.0 | -90.0 | 216.0 | -90.0 | 1102.0 | 313.0 | 12.0 | 110.0 | 720.0 | 41.0 | 720.0 | 41.0 |
| LN8 | 180.0 | 76.0 | 110.0 | 25.0 | 11539.0 | 273.0 | -166.0 | 439.0 | 208.0 | -82.0 | 217.0 | -82.0 | 1068.0 | 310.0 | 11.0 | 108.0 | 693.0 | 38.0 | 692.0 | 38.0 |
| LN9 | 352.0 | 60.0 | 126.0 | 26.0 | 12483.0 | 272.0 | -212.0 | 484.0 | 211.0 | -113.0 | 211.0 | -113.0 | 967.0 | 251.0 | 11.0 | 101.0 | 613.0 | 37.0 | 613.0 | 37.0 |
| LN10 | 418.0 | 57.0 | 126.0 | 25.0 | 12502.0 | 270.0 | -216.0 | 486.0 | 208.0 | -117.0 | 208.0 | -117.0 | 977.0 | 253.0 | 11.0 | 100.0 | 618.0 | 37.0 | 618.0 | 37.0 |
| LN11 | 354.0 | 60.0 | 126.0 | 25.0 | 12487.0 | 273.0 | -212.0 | 485.0 | 211.0 | -113.0 | 211.0 | -113.0 | 967.0 | 251.0 | 11.0 | 101.0 | 613.0 | 37.0 | 613.0 | 37.0 |
| LN12 | 273.0 | 63.0 | 129.0 | 26.0 | 12678.0 | 280.0 | -212.0 | 492.0 | 217.0 | -112.0 | 217.0 | -112.0 | 882.0 | 224.0 | 9.0 | 98.0 | 554.0 | 31.0 | 554.0 | 31.0 |
| LN13 | 262.0 | 59.0 | 132.0 | 25.0 | 13150.0 | 286.0 | -224.0 | 510.0 | 219.0 | -123.0 | 219.0 | -123.0 | 780.0 | 194.0 | 8.0 | 94.0 | 484.0 | 27.0 | 484.0 | 27.0 |
| LN14 | 388.0 | 53.0 | 133.0 | 26.0 | 13035.0 | 278.0 | -231.0 | 509.0 | 211.0 | -128.0 | 211.0 | -128.0 | 837.0 | 205.0 | 9.0 | 95.0 | 517.0 | 31.0 | 517.0 | 31.0 |
| LN15 | 176.0 | 77.0 | 119.0 | 25.0 | 12533.0 | 290.0 | -173.0 | 463.0 | 230.0 | -93.0 | 230.0 | -93.0 | 506.0 | 145.0 | 3.0 | 104.0 | 334.0 | 10.0 | 334.0 | 10.0 |
| LN16 | 295.0 | 58.0 | 130.0 | 25.0 | 13117.0 | 284.0 | -222.0 | 506.0 | 217.0 | -123.0 | 217.0 | -123.0 | 768.0 | 193.0 | 8.0 | 95.0 | 479.0 | 26.0 | 479.0 | 26.0 |
| LN17 | 239.0 | 83.0 | 132.0 | 28.0 | 12167.0 | 292.0 | -173.0 | 465.0 | 229.0 | -83.0 | 229.0 | -83.0 | 520.0 | 174.0 | 2.0 | 122.0 | 381.0 | 8.0 | 381.0 | 8.0 |
| LN18 | 348.0 | 80.0 | 128.0 | 28.0 | 11840.0 | 284.0 | -168.0 | 452.0 | 222.0 | -82.0 | 222.0 | -82.0 | 556.0 | 177.0 | 2.0 | 119.0 | 400.0 | 8.0 | 400.0 | 8.0 |
| LN19 | 570.0 | 73.0 | 120.0 | 27.0 | 11229.0 | 268.0 | -163.0 | 431.0 | 209.0 | -80.0 | 209.0 | -80.0 | 683.0 | 213.0 | 3.0 | 120.0 | 487.0 | 10.0 | 487.0 | 10.0 |
| LN20 | 145.0 | 86.0 | 119.0 | 27.0 | 11542.0 | 284.0 | -152.0 | 436.0 | 226.0 | -71.0 | 226.0 | -71.0 | 593.0 | 182.0 | 3.0 | 118.0 | 420.0 | 10.0 | 420.0 | 10.0 |
| LN21 | 534.0 | 71.0 | 121.0 | 27.0 | 11470.0 | 269.0 | -168.0 | 437.0 | 210.0 | -85.0 | 210.0 | -85.0 | 635.0 | 195.0 | 3.0 | 119.0 | 450.0 | 10.0 | 450.0 | 10.0 |
| LN22 | 202.0 | 81.0 | 117.0 | 26.0 | 11558.0 | 279.0 | -156.0 | 435.0 | 222.0 | -76.0 | 222.0 | -76.0 | 593.0 | 182.0 | 3.0 | 117.0 | 417.0 | 10.0 | 417.0 | 10.0 |
| LN23 | 52.0 | 93.0 | 110.0 | 25.0 | 11783.0 | 291.0 | -143.0 | 434.0 | 236.0 | -67.0 | 236.0 | -67.0 | 554.0 | 164.0 | 2.0 | 110.0 | 374.0 | 9.0 | 374.0 | 9.0 |
| LN24 | 21.0 | 87.0 | 108.0 | 24.0 | 12104.0 | 289.0 | -157.0 | 446.0 | 234.0 | -79.0 | 234.0 | -79.0 | 722.0 | 191.0 | 7.0 | 98.0 | 449.0 | 24.0 | 449.0 | 24.0 |
| LN25 | 27.0 | 96.0 | 81.0 | 20.0 | 10967.0 | 274.0 | -114.0 | 388.0 | 223.0 | -33.0 | 229.0 | -53.0 | 688.0 | 187.0 | 9.0 | 100.0 | 427.0 | 32.0 | 426.0 | 32.0 |
| LN26 | 389.0 | 75.0 | 134.0 | 28.0 | 12264.0 | 288.0 | -181.0 | 469.0 | 223.0 | -92.0 | 223.0 | -92.0 | 486.0 | 159.0 | 2.0 | 117.0 | 347.0 | 8.0 | 347.0 | 8.0 |
| LN27 | 739.0 | 66.0 | 122.0 | 27.0 | 11402.0 | 264.0 | -172.0 | 436.0 | 204.0 | -90.0 | 204.0 | -90.0 | 649.0 | 201.0 | 3.0 | 116.0 | 462.0 | 10.0 | 462.0 | 10.0 |
| LN28 | 396.0 | 77.0 | 119.0 | 27.0 | 11395.0 | 273.0 | -159.0 | 432.0 | 215.0 | -78.0 | 215.0 | -78.0 | 639.0 | 194.0 | 3.0 | 117.0 | 451.0 | 10.0 | 451.0 | 10.0 |
| LN29 | 248.0 | 79.0 | 116.0 | 26.0 | 11484.0 | 275.0 | -157.0 | 432.0 | 218.0 | -77.0 | 218.0 | -77.0 | 602.0 | 183.0 | 3.0 | 115.0 | 421.0 | 10.0 | 421.0 | 10.0 |
| LN30 | 122.0 | 87.0 | 110.0 | 24.0 | 12044.0 | 290.0 | -154.0 | 444.0 | 234.0 | -76.0 | 234.0 | -76.0 | 568.0 | 161.0 | 3.0 | 105.0 | 371.0 | 12.0 | 371.0 | 12.0 |
| LN31 | 105.0 | 88.0 | 107.0 | 24.0 | 11886.0 | 288.0 | -149.0 | 437.0 | 233.0 | -73.0 | 233.0 | -73.0 | 581.0 | 166.0 | 3.0 | 106.0 | 380.0 | 12.0 | 380.0 | 12.0 |
| LN32 | 69.0 | 83.0 | 109.0 | 24.0 | 12106.0 | 286.0 | -161.0 | 447.0 | 231.0 | -82.0 | 231.0 | -82.0 | 749.0 | 198.0 | 7.0 | 99.0 | 467.0 | 25.0 | 467.0 | 25.0 |
| HB1 | 552.0 | 74.0 | 130.0 | 28.0 | 11744.0 | 284.0 | -169.0 | 453.0 | 216.0 | -86.0 | 216.0 | -86.0 | 492.0 | 149.0 | 2.0 | 115.0 | 352.0 | 8.0 | 352.0 | 8.0 |
| HB2 | 684.0 | 70.0 | 120.0 | 27.0 | 11263.0 | 266.0 | -165.0 | 431.0 | 207.0 | -83.0 | 207.0 | -83.0 | 681.0 | 215.0 | 4.0 | 119.0 | 488.0 | 12.0 | 488.0 | 12.0 |
| HB3 | 760.0 | 66.0 | 120.0 | 27.0 | 11244.0 | 262.0 | -169.0 | 431.0 | 202.0 | -88.0 | 202.0 | -88.0 | 686.0 | 215.0 | 4.0 | 117.0 | 489.0 | 12.0 | 489.0 | 12.0 |
| HB4 | 552.0 | 74.0 | 130.0 | 28.0 | 11744.0 | 284.0 | -169.0 | 453.0 | 216.0 | -86.0 | 216.0 | -86.0 | 492.0 | 149.0 | 2.0 | 115.0 | 352.0 | 8.0 | 352.0 | 8.0 |
| JL1 | 516.0 | 46.0 | 128.0 | 25.0 | 12928.0 | 266.0 | -235.0 | 501.0 | 201.0 | -135.0 | 201.0 | -135.0 | 912.0 | 227.0 | 10.0 | 95.0 | 565.0 | 35.0 | 565.0 | 35.0 |
| JL2 | 408.0 | 54.0 | 127.0 | 25.0 | 12776.0 | 272.0 | -223.0 | 495.0 | 208.0 | -125.0 | 208.0 | -125.0 | 930.0 | 234.0 | 11.0 | 96.0 | 578.0 | 37.0 | 578.0 | 37.0 |
| JL3 | 390.0 | 51.0 | 128.0 | 25.0 | 12948.0 | 272.0 | -229.0 | 501.0 | 207.0 | -130.0 | 207.0 | -130.0 | 890.0 | 221.0 | 10.0 | 94.0 | 550.0 | 35.0 | 550.0 | 35.0 |
| HLJ | 319.0 | -2.0 | 133.0 | 23.0 | 15682.0 | 259.0 | -306.0 | 565.0 | 186.0 | -218.0 | 186.0 | -218.0 | 501.0 | 141.0 | 3.0 | 108.0 | 332.0 | 11.0 | 332.0 | 11.0 |
| NMG1 | 168.0 | 62.0 | 126.0 | 25.0 | 13299.0 | 290.0 | -204.0 | 494.0 | 223.0 | -119.0 | 223.0 | -119.0 | 410.0 | 123.0 | 2.0 | 109.0 | 283.0 | 8.0 | 283.0 | 8.0 |
| NMG2 | 978.0 | 10.0 | 134.0 | 27.0 | 13339.0 | 248.0 | -248.0 | 496.0 | 174.0 | -169.0 | 174.0 | -169.0 | 428.0 | 136.0 | 2.0 | 117.0 | 309.0 | 8.0 | 309.0 | 8.0 |

The recovered environmental workbook contained additional monthly precipitation/temperature and site descriptors. To avoid redundancy and multicollinearity, the confounder analysis used the 19 BIOCLIM variables shown here, reduced by PCA, plus altitude.

# **Table S14. Broader 69-site resource survey used for geographic context**

| **Survey_ID** | **Location** | **Survey_type** | **Latitude** | **Longitude** | **Altitude_m** | **Community_type** |
| --- | --- | --- | --- | --- | --- | --- |
| P1 | 辽宁省朝阳市北票市 | Wild habitat | 41.77 | 121.16 | 488.9 | 灌丛 |
| P2 | 辽宁省朝阳市朝阳县 | Wild habitat | 41.71 | 120.3 | 508.7 | 灌丛 |
| P3 | 辽宁省朝阳市建平县 | Wild habitat | 41.54 | 119.52 | 775.7 | 针叶林 |
| P4 | 辽宁省朝阳市喀左县 | Wild habitat | 41.21 | 119.61 | 536.8 | 灌丛 |
| P5 | 辽宁省朝阳市喀左县 | Wild habitat | 41.42 | 119.81 | 401.5 | 灌丛 |
| P6 | 辽宁省朝阳市喀左县 | Wild habitat | 41.49 | 119.88 | 726.4 | 灌丛 |
| P7 | 辽宁省朝阳市凌源县 | Wild habitat | 41.34 | 119.42 | 501.0 | 针叶林 |
| P8 | 辽宁省朝阳市凌源县 | Wild habitat | 40.8 | 118.93 | 779.8 | 灌丛 |
| P9 | 辽宁省朝阳市双塔区 | Wild habitat | 41.54 | 120.48 | 274.2 | 灌丛 |
| P10 | 辽宁省朝阳市双塔区 | Wild habitat | 41.57 | 120.55 | 302.0 | 灌丛 |
| P11 | 辽宁省葫芦岛市建昌县 | Wild habitat | 40.63 | 119.95 | 222.3 | 灌丛 |
| P12 | 辽宁省葫芦岛市建昌县 | Wild habitat | 41.05 | 120.07 | 405.9 | 灌丛 |
| P13 | 辽宁省葫芦岛市建昌县 | Wild habitat | 40.79 | 119.74 | 618.6 | 灌丛 |
| P14 | 辽宁省葫芦岛市建昌县 | Wild habitat | 40.67 | 119.81 | 428.0 | 灌丛 |
| P15 | 辽宁省葫芦岛市建昌县 | Wild habitat | 40.73 | 119.71 | 514.4 | 灌丛 |
| P16 | 辽宁省葫芦岛市建昌县 | Wild habitat | 40.73 | 119.56 | 494.7 | 阔叶林 |
| P17 | 辽宁省葫芦岛市建昌县 | Wild habitat | 40.72 | 119.56 | 525.2 | 灌丛 |
| P18 | 辽宁省葫芦岛市建昌县 | Wild habitat | 40.68 | 119.57 | 550.3 | 阔叶林 |
| P19 | 辽宁省葫芦岛市建昌县 | Wild habitat | 40.84 | 120.24 | 274.1 | 针叶林 |
| P20 | 辽宁省葫芦岛市兴城市 | Wild habitat | 40.75 | 120.4 | 245.7 | 灌丛 |
| P21 | 辽宁省锦州市北镇市 | Wild habitat | 41.72 | 121.73 | 275.0 | 阔叶林 |
| P22 | 辽宁省锦州市北镇市 | Wild habitat | 41.58 | 121.68 | 120.4 | 针叶林 |
| P23 | 辽宁省锦州市古塔区 | Wild habitat | 41.16 | 121.05 | 154.4 | 灌丛 |
| P24 | 辽宁省锦州市凌海市 | Wild habitat | 41.39 | 121.6 | 174.3 | 灌丛 |
| P25 | 辽宁省锦州市凌海市 | Wild habitat | 41.23 | 121.12 | 183.8 | 灌丛 |
| P26 | 辽宁省辽阳市文圣区 | Wild habitat | 41.27 | 123.29 | 140.5 | 灌丛草甸 |
| P27 | 河北省承德市宽城县 | Wild habitat | 40.61 | 118.57 | 384.6 | 针叶林 |
| P28 | 河北省承德市宽城县 | Wild habitat | 40.55 | 118.75 | 806.9 | 灌丛 |
| P29 | 河北省承德市宽城县 | Wild habitat | 40.54 | 118.54 | 463.3 | 灌丛 |
| P30 | 河北省承德市隆化县 | Wild habitat | 41.34 | 117.39 | 842.9 | 灌丛 |
| P31 | 河北省承德市隆化县 | Wild habitat | 41.34 | 117.65 | 582.6 | 针阔混交林 |
| P32 | 河北省承德市隆化县 | Wild habitat | 41.32 | 117.69 | 613.9 | 草甸 |
| P33 | 河北省承德市双桥区 | Wild habitat | 40.86 | 117.96 | 585.4 | 草甸 |
| P34 | 河北省承德市围场县 | Wild habitat | 41.95 | 117.78 | 1008.9 | 针叶林 |
| P35 | 河北省秦皇岛市青龙县 | Wild habitat | 40.46 | 119.33 | 328.9 | 针叶林 |
| P36 | 内蒙古自治区通辽市 | Wild habitat | 45.15 | 119.81 | 970.1 | 草甸 |
| P37 | 内蒙古自治区通辽市 | Wild habitat | 45.02 | 120.45 | 670.2 | 草甸 |
| P38 | 内蒙古自治区通辽市 | Wild habitat | 45.0 | 120.47 | 655.0 | 草甸 |
| P39 | 内蒙古自治区通辽市 | Wild habitat | 45.23 | 120.49 | 641.2 | 草甸 |
| P1 | 辽宁省海城市接文镇 | Cultivation base | 40.71 | 123.0 | 144.0 |  |
| P2 | 辽宁省海城市马风镇 | Cultivation base | 40.8 | 122.99 | 130.2 |  |
| P3 | 辽宁省鞍山市岫岩县 | Cultivation base | 40.29 | 123.59 | 57.9 |  |
| P4 | 辽宁省凤城市白旗镇 | Cultivation base | 40.32 | 123.84 | 64.0 |  |
| P5 | 辽宁省丹东市宽甸县 | Cultivation base | 40.68 | 124.78 | 249.6 |  |
| P6 | 辽宁省本溪市桓仁县 | Cultivation base | 41.39 | 125.09 | 387.3 |  |
| P7 | 辽宁省本溪市桓仁县 | Cultivation base | 41.4 | 125.03 | 400.3 |  |
| P8 | 辽宁省抚顺市新宾县 | Cultivation base | 41.71 | 124.83 | 213.5 |  |
| P9 | 辽宁省抚顺市清原县 | Cultivation base | 42.36 | 124.96 | 262.0 |  |
| P10 | 辽宁省抚顺市清原县 | Cultivation base | 42.11 | 125.08 | 409.7 |  |
| P11 | 辽宁省阜新市阜蒙县 | Cultivation base | 42.32 | 121.91 | 211.3 |  |
| P12 | 辽宁省铁岭市开原市 | Cultivation base | 42.42 | 124.8 | 307.9 |  |
| P13 | 辽宁省朝阳市朝阳县 | Cultivation base | 41.37 | 120.1 | 272.0 |  |
| P14 | 辽宁省朝阳市喀左县 | Cultivation base | 41.17 | 119.78 | 326.4 |  |
| P15 | 辽宁省葫芦岛市建昌县 | Cultivation base | 40.76 | 120.13 | 141.5 |  |
| P16 | 辽宁省葫芦岛市建昌县 | Cultivation base | 40.78 | 119.74 | 547.2 |  |
| P17 | 辽宁省葫芦岛市建昌县 | Cultivation base | 40.75 | 119.86 | 250.8 |  |
| P18 | 辽宁省葫芦岛市连山区 | Cultivation base | 40.94 | 120.51 | 149.2 |  |
| P19 | 辽宁省葫芦岛市兴城市 | Cultivation base | 40.78 | 120.36 | 201.5 |  |
| P20 | 辽宁省锦州市义县 | Cultivation base | 41.39 | 121.27 | 47.8 |  |
| P21 | 辽宁省辽阳市文圣区 | Cultivation base | 41.26 | 123.14 | 21.0 |  |
| P22 | 辽宁省大连市普兰店区 | Cultivation base | 39.64 | 122.42 | 59.7 |  |
| P23 | 吉林省通化市通化县 | Cultivation base | 41.55 | 125.55 | 418.0 |  |
| P24 | 吉林省通化市通化县 | Cultivation base | 41.71 | 125.7 | 539.0 |  |
| P25 | 河北省承德市隆化县 | Cultivation base | 41.32 | 117.7 | 546.2 |  |
| P26 | 河北省承德市宽城县 | Cultivation base | 40.6 | 118.41 | 364.6 |  |
| P27 | 河北省承德市宽城县 | Cultivation base | 40.55 | 118.76 | 683.7 |  |
| P28 | 黑龙江省黑河市嫩江市 | Cultivation base | 49.17 | 125.35 | 345.0 |  |
| P29 | 内蒙古自治区赤峰市 | Cultivation base | 42.44 | 119.06 | 278.5 |  |
| P30 | 内蒙古自治区通辽市 | Cultivation base | 43.68 | 122.49 | 177.8 |  |

The 69-site survey and the 42 analytical samples are related field resources but are not a one-to-one site list. The analytical subset was selected purposively as described in Section 2.1.

# **Table S15. Complete 91 soil-marker Spearman correlations**

| **Soil variable** | **Phytochemical marker** | **Spearman rho** | **P value** | **BH-FDR q** |
| --- | --- | --- | --- | --- |
| Organic matter | Atractylon | 0.4361 | 0.003887 | 0.194061 |
| Alkali-N | Hinesol | 0.4321 | 0.004265 | 0.194061 |
| Alkali-N | Atractylon | 0.3691 | 0.016153 | 0.489978 |
| Mo | Atractylenolide I | 0.3467 | 0.024488 | 0.557112 |
| Alkali-N | Atractylodin | 0.3170 | 0.040804 | 0.601868 |
| Available K | β-Eudesmol | 0.3102 | 0.045556 | 0.601868 |
| pH | β-Eudesmol | 0.3092 | 0.046298 | 0.601868 |
| Organic matter | Atractylodin | 0.2914 | 0.061148 | 0.625794 |
| Available K | Hinesol | 0.2906 | 0.061892 | 0.625794 |
| Alkali-N | β-Eudesmol | 0.2513 | 0.108453 | 0.806115 |
| Ca | Atractylodin | 0.2430 | 0.120955 | 0.806115 |
| Organic matter | Hinesol | 0.2410 | 0.124182 | 0.806115 |
| pH | Hinesol | 0.2334 | 0.136897 | 0.806115 |
| Mo | Atractylodin | 0.2262 | 0.149788 | 0.806115 |
| Mo | 5-HMF | 0.2241 | 0.153643 | 0.806115 |
| Mo | Hinesol | 0.2226 | 0.156532 | 0.806115 |
| pH | Atractylenolide III | -0.2206 | 0.160276 | 0.806115 |
| Zn | β-Eudesmol | -0.2142 | 0.173218 | 0.806115 |
| Available K | Atractylenolide III | -0.2084 | 0.185452 | 0.806115 |
| Ca | Atractylenolide I | 0.2049 | 0.192944 | 0.806115 |
| Alkali-N | 5-HMF | 0.2031 | 0.197094 | 0.806115 |
| Organic matter | Atractylenolide III | 0.2020 | 0.199532 | 0.806115 |
| Mn | β-Eudesmol | 0.2002 | 0.203743 | 0.806115 |
| Fe | β-Eudesmol | 0.1908 | 0.226052 | 0.815514 |
| Cu | Atractylodin | 0.1858 | 0.238732 | 0.815514 |
| Available P | Atractylon | -0.1839 | 0.243566 | 0.815514 |
| Mo | Atractylenolide III | 0.1782 | 0.258897 | 0.815514 |
| Ca | β-Eudesmol | 0.1774 | 0.261107 | 0.815514 |
| pH | Atractylodin | 0.1744 | 0.269344 | 0.815514 |
| Cu | Atractylenolide III | -0.1737 | 0.271264 | 0.815514 |
| Fe | Atractylodin | 0.1688 | 0.285353 | 0.815514 |
| Available P | Atractylenolide I | -0.1584 | 0.316299 | 0.815514 |
| Mg | Atractylodin | 0.1564 | 0.322733 | 0.815514 |
| Available B | Atractylon | 0.1556 | 0.325228 | 0.815514 |
| Mg | Atractylenolide I | 0.1540 | 0.330279 | 0.815514 |
| Fe | Atractylon | -0.1528 | 0.333917 | 0.815514 |
| Organic matter | 5-HMF | 0.1507 | 0.340663 | 0.815514 |
| Available K | Atractylenolide I | -0.1498 | 0.343665 | 0.815514 |
| Available K | Atractylodin | 0.1480 | 0.349506 | 0.815514 |
| Available P | 5-HMF | 0.1380 | 0.383421 | 0.862548 |
| Mo | β-Eudesmol | 0.1358 | 0.391233 | 0.862548 |
| Organic matter | Atractylenolide I | 0.1323 | 0.403480 | 0.862548 |
| Mg | Atractylon | -0.1312 | 0.407578 | 0.862548 |
| Zn | Atractylenolide III | -0.1265 | 0.424777 | 0.865202 |
| Mn | Atractylodin | 0.1246 | 0.431901 | 0.865202 |
| pH | 5-HMF | 0.1231 | 0.437355 | 0.865202 |
| Ca | Atractylenolide III | -0.1161 | 0.463933 | 0.885407 |
| Ca | Atractylon | 0.1129 | 0.476637 | 0.885407 |
| Available B | β-Eudesmol | -0.1128 | 0.476758 | 0.885407 |
| Zn | Hinesol | -0.1037 | 0.513336 | 0.914467 |
| Alkali-N | Atractylenolide I | 0.1036 | 0.513645 | 0.914467 |
| Mn | Atractylenolide III | -0.1015 | 0.522553 | 0.914467 |
| Ca | Hinesol | 0.0960 | 0.545239 | 0.936164 |
| Fe | 5-HMF | 0.0906 | 0.568075 | 0.957312 |
| Zn | 5-HMF | 0.0870 | 0.583708 | 0.959508 |
| Available B | 5-HMF | -0.0831 | 0.600832 | 0.959508 |
| Available P | Atractylodin | 0.0812 | 0.609213 | 0.959508 |
| Fe | Atractylenolide III | -0.0757 | 0.633761 | 0.959508 |
| Cu | Hinesol | 0.0718 | 0.651221 | 0.959508 |
| Available P | Atractylenolide III | 0.0690 | 0.664293 | 0.959508 |
| pH | Atractylenolide I | 0.0563 | 0.723139 | 0.959508 |
| Mg | Atractylenolide III | -0.0558 | 0.725797 | 0.959508 |
| Ca | 5-HMF | -0.0548 | 0.730419 | 0.959508 |
| Cu | 5-HMF | 0.0533 | 0.737497 | 0.959508 |
| Cu | Atractylon | -0.0514 | 0.746424 | 0.959508 |
| Available P | β-Eudesmol | 0.0489 | 0.758228 | 0.959508 |
| Available B | Atractylenolide III | 0.0469 | 0.768029 | 0.959508 |
| Available B | Atractylodin | -0.0460 | 0.772534 | 0.959508 |
| Available P | Hinesol | -0.0453 | 0.775758 | 0.959508 |
| Cu | β-Eudesmol | 0.0434 | 0.784726 | 0.959508 |
| Mn | Hinesol | -0.0419 | 0.792211 | 0.959508 |
| Available B | Atractylenolide I | 0.0366 | 0.818186 | 0.959508 |
| Mn | 5-HMF | 0.0359 | 0.821415 | 0.959508 |
| Alkali-N | Atractylenolide III | 0.0357 | 0.822216 | 0.959508 |
| Mg | 5-HMF | 0.0339 | 0.831350 | 0.959508 |
| Cu | Atractylenolide I | 0.0327 | 0.837097 | 0.959508 |
| Zn | Atractylenolide I | 0.0307 | 0.847183 | 0.959508 |
| Available K | 5-HMF | 0.0283 | 0.858829 | 0.959508 |
| Mo | Atractylon | -0.0268 | 0.866382 | 0.959508 |
| Available B | Hinesol | -0.0258 | 0.871264 | 0.959508 |
| pH | Atractylon | 0.0244 | 0.878142 | 0.959508 |
| Mn | Atractylon | 0.0224 | 0.888191 | 0.959508 |
| Mg | Hinesol | -0.0217 | 0.891417 | 0.959508 |
| Organic matter | β-Eudesmol | -0.0209 | 0.895446 | 0.959508 |
| Mn | Atractylenolide I | 0.0207 | 0.896243 | 0.959508 |
| Mg | β-Eudesmol | 0.0186 | 0.907144 | 0.959884 |
| Available K | Atractylon | 0.0124 | 0.937863 | 0.961583 |
| Fe | Hinesol | 0.0119 | 0.940317 | 0.961583 |
| Zn | Atractylon | 0.0088 | 0.955695 | 0.961583 |
| Fe | Atractylenolide I | -0.0083 | 0.958566 | 0.961583 |
| Zn | Atractylodin | 0.0077 | 0.961583 | 0.961583 |
